# Microglia-derived exosomes modulate myelin regeneration via miR-615-5p/MYRF axis

**DOI:** 10.1186/s12974-024-03019-5

**Published:** 2024-01-22

**Authors:** Xiao-Yu Ji, Yu-Xin Guo, Li-Bin Wang, Wen-Cheng Wu, Jia-Qi Wang, Jin He, Rui Gao, Javad Rasouli, Meng-Yuan Gao, Zhen-Hai Wang, Dan Xiao, Wei-Feng Zhang, Bogoljub Ciric, Yuan Zhang, Xing Li

**Affiliations:** 1 National Engineering Laboratory for Resource Development of Endangered Crude Drugs in Northwest China, The Key Laboratory of Medicinal Resources and Natural Pharmaceutical Chemistry, The Ministry of Education, College of Life Sciences, Shaanxi Normal University, Xi’an, 710119 Shaanxi China; 2 The Nervous System Disease Diagnosis and Treatment Engineering Technology Research Center of Ningxia, Yinchuan, 750001 China; 3 Department of Neurology, Thomas Jefferson University, Philadelphia, PA 19107 USA; 4 Department of Burns and Cutaneous Surgery, Xijing Hospital, Air Force Medical University, Xi’an, 710032 China

**Keywords:** Demyelinating disease, OPCs, Microglia, Exosomes, miR-615-5p

## Abstract

Demyelination and failure of remyelination in the central nervous system (CNS) characterize a number of neurological disorders. Spontaneous remyelination in demyelinating diseases is limited, as oligodendrocyte precursor cells (OPCs), which are often present in demyelinated lesions in abundance, mostly fail to differentiate into oligodendrocytes, the myelinating cells in the CNS. In addition to OPCs, the lesions are assembled numbers of activated resident microglia/infiltrated macrophages; however, the mechanisms and potential role of interactions between the microglia/macrophages and OPCs are poorly understood. Here, we generated a transcriptional profile of exosomes from activated microglia, and found that miR-615-5p was elevated. miR-615-5p bound to 3′UTR of myelin regulator factor (MYRF), a crucial myelination transcription factor expressed in oligodendrocyte lineage cells. Mechanistically, exosomes from activated microglia transferred miR-615-5p to OPCs, which directly bound to MYRF and inhibited OPC maturation. Furthermore, an effect of AAV expressing miR-615-5p sponge in microglia was tested in experimental autoimmune encephalomyelitis (EAE) and cuprizone (CPZ)-induced demyelination model, the classical mouse models of multiple sclerosis. miR-615-5p sponge effectively alleviated disease progression and promoted remyelination. This study identifies miR-615-5p/MYRF as a new target for the therapy of demyelinating diseases.

**Supplementary Information:**

The online version contains supplementary material available at 10.1186/s12974-024-03019-5.

## Introduction

Multiple sclerosis (MS) is a demyelinating disease of the central nervous system (CNS), characterized by failure of myelin repair, neuronal loss, and accumulation of neurologic deficit. Experimental autoimmune encephalomyelitis (EAE) and cuprizone (CPZ)-induced demyelination model are commonly used animal model of MS [1, 2]. Oligodendrocyte precursor cells (OPCs) constitute a heterogeneous population that resides in the brain parenchyma since embryonic development [3]. The physiological function of OPCs is to differentiate into mature oligodendrocytes (OLGs), myelinate axons, and promote the transmission of nerve impulses. Although myelination is the primary function of OLGs, additional roles of these cells have been identified, such as nutritional support for neurons, phagocytosis, and even antigen presentation [4, 5]. In the initial stages of MS, remyelination is mediated by OPC development [6]. Following acute demyelination in the CNS, OPCs respond to changes in the microenvironment, rapidly proliferate and migrate to the injury site, and differentiate into mature OLGs, thereby gradually restoring myelin sheaths and neurotransmission [7]. However, the hostile microenvironment in MS lesions eventually weakens OPC differentiation to OLGs and remyelination process [8]. Microglia are CNS resident macrophages. In steady state, microglia are quiescent and engaged in homeostatic functions, but if homeostasis is disrupted, such as by inflammation, microglia become activated and exhibit additional functions. For example, in MS, when microglia are stimulated by infiltrating inflammatory cells, they become antigen presenting cells and, among other things, secrete large quantities of inflammatory cytokines and chemokines [9]. It is believed that the inflammatory factors released by microglia aggravate the damage to OLGs and myelin sheaths [10], but whether activated microglia have other effects on myelin, and how intercellular communication with OPCs occurs is still poorly understood.

Exosomes are nanoscale vesicles that almost all types of cells can release. Exosomes transport various biologically active molecules, such as proteins, mRNA, microRNA (miRNA), DNA, and lipids among cells to regulate their physiological functions [11]. Exosomes are important in cell–cell communication, including in the regulation of synaptic activity, myelin biogenesis, and repair of damaged neurons [12]. However, relatively little is known about how exosome-mediated communication among glial cells regulates the function of OPCs after demyelination.

In the present study, we found that exosomes from activated microglia inhibited the differentiation of OPCs to OLGs and remyelination. It has been mentioned previously, above profiling showed that miR-615-5p was enriched in the exosomes. Bioinformatics analysis and luciferase assay demonstrated that MYRF is the target of miR-615-5p. EAE mice and CPZ mice treated with AAV that expresses miR-615-5p sponge in microglia had alleviated clinical disease, accompanied with enhanced OLGs maturation and remyelination. Our study defined a new mode and role of microglia–OPC communication in inflamed CNS; these findings could provide a foundation for novel approaches to treat MS.

## Material and methods

### EAE induction

Female C57BL/6 mice, 8 weeks of age, were purchased from the Experimental Animal Center, Shaanxi Normal University. Each mouse was first anesthetized with 200 µl pentobarbital sodium (concentration: 5 mg/mL), followed by an intraperitoneal injection of 200 ng pertussis toxin (PTX, List Lab). Then, the emulsified antigen MOG_35-55_ (Genscript) was injected into two subcutaneous positions in the middle back of each mouse’s forelimb and hindlimb trunk, 200 μg for each mouse. Next, complete Freund’s adjuvant (CFA) was prepared by mixing incomplete Freund’s adjuvant (Sigma) with 10 mg/ml of *M. tuberculosis Des.* H37 Ra (BD Biosciences), and 100 μL CFA were combined with 100 μL MOG_35-55_ working solution and injected into each mouse [13]. After 48 h of immunization, mice were intraperitoneally injected with 200 ng PTX. Clinical EAE was scored daily in a blind manner, according to a 0–5 scale as described previously: 0, no clinical signs; 0.5, stiff tail; 1, limp tail; 1.5, limp tail and wadding gait; 2, paralysis of one limb; 2.5, paralysis of one limb and weakness of another limb; 3, complete paralysis of both hind limbs; 4, moribund; and 5, death [14].

### CPZ treatment

Male C57BL/6 mice, 8 weeks of age, were purchased from the Experimental Animal Center, Shaanxi Normal University. To induce demyelination of the CNS without substantial immunoreactivity, mice were fed standard rodent chow containing 0.2% CPZ for 5 weeks. The naïve group was fed a standard diet.

### Behavioral tests

*Tightrope test*. The two ends of a cotton rope with a diameter of 2 mm were tautly placed on two platforms with a height of 60 cm and an interval of 50 cm between the two platforms. During the experiment, the mice were placed in the middle of the tightrope, and the time taken by the mice from the middle of the tightrope to the platform of either end was recorded. Each mouse was subjected to three experiments, each with an interval of 60 s. The final result was taken as the average of the three times.

*Fatigue baton twister test*. Mice were trained to maintain balance and locomotion on the fatigue baton twister before the start of the experiment and were acclimatized at 5 rpm for 5 min. During the investigation, the mice were placed on the baton twister with the baton twister at an initial speed of 5 rpm and accelerated at an increase of 1 rpm per second until the mice dropped, and the time the mice spent on the baton twister with the baton locomotion was recorded. Each mouse was subjected to two experiments with an interval of 60 s between the two experiments. The final result was taken as the average of the 2 experiments. After each experiment, the apparatus was cleaned with 75% ethanol to prevent interference with the following mouse experiment.

### Cell culture and treatment

Macrophage cell line Raw264.7 is cultured in a complete medium containing DMEM (Corning), 10% FBS (ExCell Bio), and 1% Pen Strep (Gibco). The spontaneously immortalized mouse microglia line SIM-A9 (SM cells) retains critical attributes of primary microglia [15] and is cultured in a complete medium containing DMEM/F12 (Corning), 10% fetal bovine serum (FBS) (ExCell Bio), 5% horse serum (HS) (Gibco) and 1% penicillin–streptomycin (Pen Strep). SM cells were divided into two groups: both groups of cells were cultured in the complete medium until the cell density reached 80%. At this time, cells were placed either in a fresh complete medium, in a complete medium containing LPS (100 ng/mL), or in a complete medium containing LPS (100 ng/mL) and miR-615-5p inhibitor (100 nM). After 18 h of culture, the cells were placed in a fresh complete medium and cultured for another 24 h, and the culture supernatant was then collected.

Primary microglia and OPCs were isolated from newborn mouse brain (P3) by dissociation with Neural Tissue Dissociation Kit (Miltenyi Biotech Inc.) and purification with either anti-CD11b or anti-CD140a microbeads (Miltenyi Biotec Inc.), respectively. Primary microglia were cultured in DMEM (Gibco), 10% FBS (Corning), 1% GlutaMAX, 1% Pen Strep, and 5 ng/ml M-CSF cell culture medium, while OPCs were cultured in DMEM/F12 (Gibco) supplemented with 2% B27 (Gibco), 1% N_2_ (Gibco), and 50% B104 supernatant. After 3 days of cell culture, the primary microglia and OPCs were activated with LPS (100 ng/ml) for 18 h, and their RNA was collected.

B104 neuroblastoma cell, a neuronal cell line, was gifted by Prof. Zeng-Qiang Yuan (Beijing Institute of Basic Medical Sciences). B104 cells were first cultured in a proliferation medium containing DMEM/F12 basal medium (Gibco), 10% FBS (Corning), and 1% Pen Strep. When cell density reached 80%, B104 cells were washed twice with Hank's Balanced Salt Solution (HyClone) and then cultured with a conditioned medium for 48 h. The conditioned medium contained DMEM basal medium (Gibco), 1% GlutaMax (Gibco), and 1% Pen Strep. It has been documented that B104 supernatant collected after the replacement of a conditioned medium can promote the growth of OPCs [16]. Thus, the B104 supernatant collected after 48 h of culture was used as one of the components of the culture medium for primary OPCs.

### EXOs isolation

EXOs were isolated from the supernatant of SM cells. FBS and HS for culturing SM cells were ultracentrifuged at 110, 000 × *g* for 3 h at 4 ℃ to deplete FBS exosomes [17]. The supernatant was harvested and filtered through the 0.22-μm filter (Millipore), and SM-derived EXOs or LPS pretreated SM-derived EVs (EXOs-LPS) were extracted by ultracentrifugation. Briefly, cell supernatant was centrifuged at 300×*g* for 10 min to remove cells; 2000×*g* for 20 min, and 8000×*g* for 30 min to remove cell debris [18]. Finally, the medium was centrifuged at 110, 000×*g* for 70 min at 4 ℃ in an SW40-Ti rotor and Optima XPN-100 ultracentrifuge (Beckman). The supernatant was removed, and the EXOs were washed with PBS and ultracentrifuged again at 110,000×*g* for 70 min at 4 ℃ to remove impurities further. Finally, the EXOs or EXOs-LPS pellet was suspended with PBS and stored at − 80 °C.

### Exoview

EXOs and EXOs-LPS were captured with an Exosome detection kit (NanoView Biosciences), which was coated with exosomal antibodies against CD81 and CD9. IgG isotype was used as a negative control. After capturing EXOs or EXOs-LPS, the Exoview Tetraspanin Chips were incubated with anti-CD63 and anti-CD9 to characterize exosomes. Biotinylated miR-615-5p probe (GenePharma) was mixed with SA-Cy3 (streptavidin-Cy3; GenePharma) to detection EXOs or EXOs-LPS containing miR-615-5p. After denaturation of the probe at 75 ℃ for 10 min, the probe solution was incubated with the chip overnight. Finally, the chip was imaged using the ExoView R100 Reader, and the data were analyzed using the ExoViewer 2.5.0.

### Nanoparticle tracking analysis (NTA)

The concentration and particle size of the isolated EXOs were measured by Zetaview-PMX120-Z (Particle Metrix). The mean particle size, distribution, and amounts were analyzed by Zetaview software (version 8. 05. 14 SP7).

### Transmission electron microscopy (TEM)

The morphology of EXOs was observed by transmission electron microscopy (TEM). Briefly, EXOs were resuspended in PBS and fixed with 2% paraformaldehyde for 30 min at room temperature (RT). Subsequently, 2 μL of the mixture was added to the carbon film copper grids for 10 min at RT. Adherent EXOs were stained with uranyl acetate and immediately observed under the electron microscope.

The remyelination effect of the spinal cord was evaluated by TEM. After 27 days of immunization, mice were perfused with PBS, and the lumbar spinal cords was harvested to prepare TEM samples. The samples were fixed in 2.5% glutaraldehyde for 24 h, postfixed in 1% OsO4 for 4–6 h, and stained with 1% uranyl acetate. The spinal cord slices were then dehydrated in ethanol and infiltrated with Epon using propylene oxide as a transition solvent. The Epon was polymerized at 60 °C for 48 h and sectioned on an ultramicrotome. Remyelinated axons were defined by the g ratio, the axon diameter divided by the entire fiber diameter of that axon plus the myelin sheath. At least 50 myelinated or demyelinated axons on the TEM micrographs were determined for each mouse.

### Western blot

Anti-MYRF (Thermo, PA5-113,555, 1:5000), anti-MBP (Thermo, PA1-10,008, 1:5000), anti-β-actin (Invitrogen, MA1-140, 1:4000) were analyzed by western blot. Briefly, cells or tissue were lysed by RIPA lysis buffer. Then, the extracted protein was mixed with a loading buffer and used for polyacrylamide gel electrophoresis. Based on the BCA protein quantitation method, the same quality protein is used between the different groups. Finally, the gel was incubated with a polyvinylidene fluoride (PVDF) membrane (Millipore) to transfer the protein. Then the PVDF membrane was blocked with Tris-buffered saline with Tween-20 fat-free dried milk (BD Biosciences) at RT for 1–3 h and incubated with primary antibodies at 4 ℃ overnight, further incubated with secondary antibodies at RT for 1–2 h. Finally, the protein was observed in a chemiluminescence analysis system (Tanon).

### Patients and tissue samples

Brain tissue from MS patients was purchased from the Rocky Mountain MS Center (Aurora, Colorado). This work was authorized by the Institutional Review Board of Thomas Jefferson University (TJU). The postmortem tissues of MS patients were fixed with formalin and embedded in paraffin. The paraffin sections (10 μm thick) were stained by immunohistochemistry.

### Histology and immunofluorescence staining

Spinal cord tissue was fixed in 4% paraformaldehyde (PFA) in PBS and embedded in paraffin. The spinal cord sections were stained with hematoxylin and eosin (H&E) and Luxol Fast Blue (LFB) to screen for inflammation and demyelination. They are examined and evaluated using previously described methods [19].

Black gold immunostaining stains the myelin a reddish brown color, which is used to determine the integrity of the myelin [20]. The brain was fixed in 4% PFA and embedded by Tissue-Tek O.C.T. Compound (OCT) (Sakura). Then the tissues were cut into 20 μm sections and adhered to the slides. The slices were kept at 37 °C for 30 min, and stained at 45 °C in the dark for 20 min. Finally, the slices were rinsed, a staining terminator was added, and the slices were sealed with a water-soluble sealer.

The brain and spinal cord were fixed in 4% PFA and embedded by Tissue-Tek O.C.T. Compound (OCT) (Sakura) were cut into 8 μm sections and adhered to the slides. The slides were fixed in 4% PFA for 20 min. Antigen retrieval was performed in citrate at 85° in a water bath for 30 min. The slides were washed in PBS (15 min, RT, shaking). The primary antibody was diluted in the blocking buffer at 4 ℃ overnight. The secondary antibody was diluted in the blocking buffer and incubated at RT for 1 h. The tissue section was washed in PBS 3 times, and the ProLong™ Diamond antifade mountant buffer (with DAPI) (Thermo) was used to seal the samples.

The cell samples were fixed in 4% PFA at RT for 30 min. Then, the cell samples were washed 3 times in PBS. Then, the samples were treated with 0.3% Triton X-100 for 15 min. After washing with PBS three times, the primary antibody was diluted in 10% HS in PBS and incubated overnight at 4 ℃. Excess antibodies were washed 3 times in PBS. The secondary antibodies were diluted in 2% HS in PBS and incubated for 1 h at RT. Again, excess antibodies were washed 3 times in PBS. Finally, the ProLong™ Diamond antifade mountant buffer (with DAPI) was used to seal the cell coverslips.

The use of primary antibodies has Rb anti-PDGFRα (Abcam, ab203491, 1:500), Ms anti-CNP(Abcam, ab6319, 1:500), Chk anti-MBP (ThermoFisher, PA1-10,008, 1:2000), Rb anti-MYRF (OasisBiofarm, OB-RB007-02, 1:1000), Rb anti-Iba1 (Wako, 019–19741, 1:500), Ms anti-APC (Calbiochem, OP80-100UG 1:100), Ms anti-PDGFRα (Abcam, ab96569 1:500), Ms anti-GFAP (EMD Millipore, MAB360, 1:800), Rb anti-NeuN (Abcam, ab177487 1:500). The use of secondary antibodies has Donkey Anti-Chicken (JACKSON, 703–585-155, 1:800); Donkey anti-Rabbit (JACKSON, 711–585-152, 1:800), Donkey anti-Mouse (JACKSON, 715–585-150, 1:800), Goat anti-Rabbit (JACKSON, 111–545-144, 1:800), Goat anti-Mouse (JACKSON, 115–545-062, 1:800).

### Fluorescence in situ hybridization (FISH)

SM and Raw264.7 cells were added to a 24-well plate containing coverslips coated with poly-D-lysine (Sigma). When the cells multiplied to 50% density, they were divided into two groups: one group was treated with a normal medium, and another was treated with medium containing LPS for 18 h. Then, slides were fixed with 4% PFA, and the targeted sites were opened using different buffers in the DTG miRNA in situ hybridization detection kit (Guangzhou Exon Biotechnology Co., ltd). In addition, tissues were treated with 3% H_2_O_2_ in order to eliminate endogenous peroxidase. 18 s/miR-615-5p probe (Guangzhou Exon Biotechnology) was added onto the tissues and hybridized at 37 °C for 18 h. After hybridization, anti-digoxin HRP conjugate was incubated with the tissues. Finally, the color was developed with the chromogenic agent from the kit.

The tissue that detects miR-615-5p were derived from mice with an EAE clinical score of 3. The spinal cord lumbar of naïve and EAE mice was obtained at the disease peak (17 days post-immunization). Fresh tissues were fixed in 4% PFA, dehydrated in 30% sucrose, embedded in OCT (SAKURA), and cut into 8 µm sections. Similar to the process of FISH on cell slides, the tissue sections were labeled by probe hybridization, HRP conjugation, and TSA-555 chromogenesis. In addition, to identify the expression of miR-615-5p in microglia, OPCs, oligodendrocytes, astrocytes, and neurons, we combined FISH with frozen-section immunofluorescence staining to detect the co-localize of miR-615-5p and the Iba1, PDGFRα, APC, GFAP, and NeuN.

### RNA sequencing analysis

RNA was extracted from EXOs using Trizol reagent (Invitrogen) and Dr. GenTLE™ Precipitation Carrier (TaKaRa). The quality of all RNA samples was evaluated by gel electrophoresis, Nanodrop, and Agilent 2200. After passing the RNA quality test, RNA with polyA tail was enriched by polyA library construction and ribosomal library construction. The expression of miRNA was detected by the HiSeq platform at NovelBio.

### miR-615-5p knockdown by AAV

Both AAV-Iba1p-EGFP-miR-615-5p-Sponge and AAV-Iba1p-EGFP-Scramble viruses were purchased from Shanghai Genechem. miR-615-5p-Sponge and EGFP act as a tandem expression cassette driven by the Iba1 promoter and are specifically expressed in microglia. In addition, EGFP expression is regulated by a specific promoter and can express green fluorescent protein within microglia for subsequent observation after virus injection. Two days before EAE modeling and one week after CPZ modeling, all mice were injected with the AAV virus at the lateral ventricle (coordinates are: the anterior fontanelle as the center point, 1 mm to the left, 1 mm to the posterior fontanelle, and 2.5 mm depth). Each mouse was injected with 20 μL AAV-miR-615-5p-Sponge/AAV-Scramble/PBS. The rate was 2 μL/min, and the final virus dose per mouse was 1 × 10^10^ vg.

### Vector construction (luciferase/MYRF)

In order to construct the pCDH-CMV-Luc-MYRF 3′-UTR vector, the luciferase fragment was successively inserted into the multiple cloning site of pCDH-CMV-MSC-EF1-copGFP through XbaI and NotI. Then, the MYRF 3′UTR fragment was ligated to the 3′ end of luciferase via NotI and BamHI. The vector was named pCDH-CMV-Luc-MYRF 3′UTR-EF1-copGFP, and the schematic diagram is shown in Additional file 1: Fig. S1A. Similar to the previous vector construction, after the 3′UTR region of MYRF was mutated using the PrimeSTAR Max DNA Polymerase kit (TaKaRa), its mutated 3′-UTR region fragment was connected to the 3′end of Luciferase to obtain the mutant vector. The vector was named pCDH-CMV-Luc-Mut MYRF 3′UTR. Additional file 1: Fig. S1B shows the vector diagram. Sets for murine genes are listed in Additional file 1: Table S1.

### Luciferase report assays

Report plasmids contained wild-type (WT) or mutant (MUT) 3′UTR of MYRF, which were constructed and transfected into HEK293T cells. First, HEK293T cells were cultured in 96-well plates at 10^5^ cells per well for 24 h. Next, the plasmids and miR-615-5p mimics were transfected into HEK293T cells for 6 h by the transfection reagent polyethylenimine (PEI) (Polysciences). Then, the cells were placed in a complete medium, and the luciferase activity was measured after culturing for another 36 h. The 96-well plate was kept at RT, and 80 µl of assay reagent was added to the medium. After incubation at RT for 3 min, the 96-well plate was mixed at 300 rpm on a shaker for 3 min. Finally, report gene activity was measured with the one-GLo™ EX Luciferase Assay System (Promega).

### RNA extraction and qRT-PCR

Total RNA was extracted by RNA Easy Fast Kit (TIANGEN Biotech), and total RNA extracted from EVs was using Trizol reagent. Reverse transcription was conducted using the PrimeScript RT Master Mix (Perfect Real Time) (Takara Bio). qRT-PCR was performed using the Hieff^®^ qPCR SYBR Green Master Mix (No Rox) (Yeasen Biotech) in CFX96 Touch Real-Time PCR Detection System or T100 Thermal Cycler (Bio-Rad). Relative gene expression levels were normalized to GAPDH or U6 and calculated using the 2^−ΔΔt^ method. Primer sets for murine genes are listed in Additional file 1: Table S1, S2.

### Statistical analyses

The software for calculating the fluorescence intensity of MBP per unit area was Image-Pro Plus. ImageJ analyzed the bands obtained by WB for gray values. All data were analyzed using GraphPad Prism 8.0 software. Ordinary one-way ANOVA or two-way ANOVA test was performed by multiple comparisons or pairwise comparisons when comparing multiple groups. All other statistical comparisons were made using the nonparametric t-test. Data are presented as the mean ± SEM and significance were set as *P < 0.05, **P < 0.01, ***P < 0.001, ****P < 0.001.

## Results

### Microglia and OPCs accumulate in MS/EAE lesions, but OPC differentiation is blocked

**Fig. 1:**
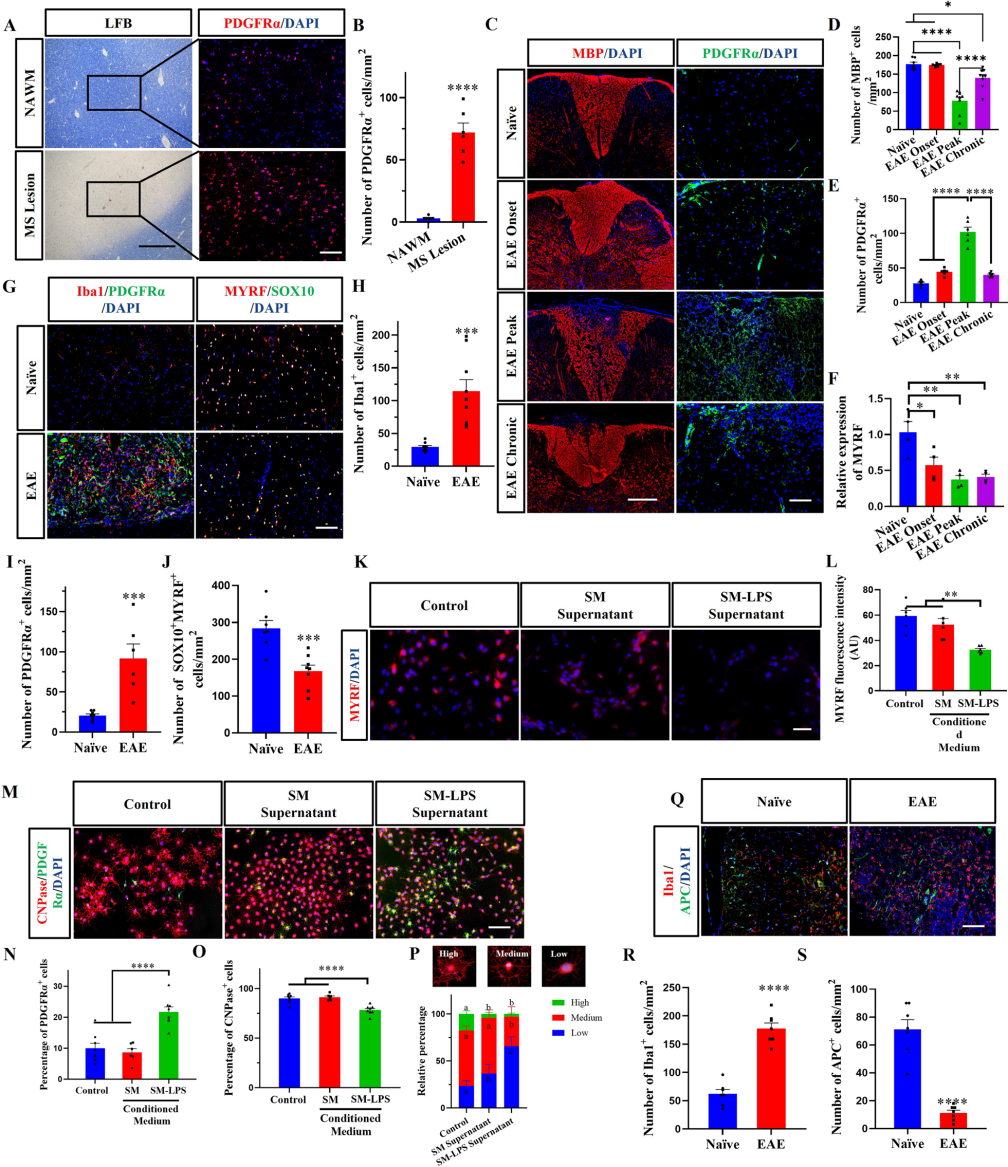
Microglia and OPCs were aggregated in the inflammatory microenvironment, and the differentiation of OPCs was inhibited. **A** Immunofluorescence staining of PDGFRα in the lesion of human brain sample, and **B** the number of PDGFRα^+^ cells per mm^2^. **C** Immunofluorescence staining of MBP and PDGFRα in spinal cords of naïve mice, and mice with EAE at disease onset (day 10 p.i.), peak (day 17 p.i.) and chronic phase (day 30 p.i.); and **D**, **E** quantitative MBP^+^ area per mm^2^ and the number of PDGFRα^+^ cells per mm^2^. **F** qRT-PCR of MYRF in spinal cords of naïve and EAE mice at different stages. **G** Immunofluorescence staining of Iba1/PDGFRα and MYRF/SOX10 (oligodendrocyte lineage cells marker) in spinal cords of naïve and EAE mice (day 17 p.i.), and **H**–**J** the number of Iba1^+^, PDGFRα^+^, SOX10^+^MYRF^+^ cells per mm^2^. **K** OPCs incubated with supernatant and immunofluorescence staining for MYRF, and **L** MYRF fluorescence intensity. **M** OPCs incubated with supernatant and immunofluorescence stained with PDGFRα and CNPase, and **N**, **O** percentage of PDGFRα^+^, CNPase^+^ cells per mm^2^. **P** The relative percentage of three differentiation stages of OPCs in each experimental group. **Q** Immunofluorescence staining of Iba1 and APC in spinal cords of naïve and EAE mice (day 17 p.i.), and **R**, **S** the number of Iba1^+^ and APC^+^ per mm^2^. Each data point represents the average of 4–6 regions of interest analyzed per section from at least 3 sections per animal with n ≥ 3 mice per group. Spinal cord samples analyzed in the white matter lesion area (**C**, **G**, **Q**). Typical representative figures show the dorsal cord lesion area of the spinal cord (**C**). All data are represented by mean ± SEM. T-test was used to determine *P* values (**B**, **H**, **I**, **J**, **R**, **S**). One-way ANOVA was used to determine *P* values (**D**, **E**, **F**, **L**, **N**, **O**). * *P* < 0.05, **P < 0.01, ***P < 0.001, ****P < 0.0001.Two-way ANOVA was used to determine *P* values (**P**). Groups that do not share the same letter are significantly (P < 0.05). One representative of three independent experiments is shown. Scale bar = 200 μm (the left part of figure **A** and **C**), 100 μm (**A**, the right part of **A** and **C**, **G**, **M**, **P**), and 50 μm (**K**)

We first confirmed that a large number of PDGFRα^+^ OPCs were accumulated in the brain lesions of MS patients, as well as in the spinal cord lesions of mice with EAE (Fig. 1A–E). Compared with the naїve mice or mice at the onset and chronic stage of EAE, the numbers of PDGFRα^+^ OPCs in the demyelinated areas (MBP^−^) were increased at disease peak (Fig. 1C–E). These data suggested that OPCs accumulate in the lesions in large numbers but do not differentiate into OLGs.

MYRF is a critical transcription factor for myelination, and it is directly related to OPCs differentiation [21, 22]. We found that the relative expression of MYRF mRNA was reduced at the peak of EAE (Fig. 1F). In addition, the accumulation of OPCs in the lesions was accompanied by the accumulation of Iba1^+^ microglia/macrophage (Fig. 1G–I). A large number of Iba1^+^ microglia/macrophage cells were also observed in the MBP^−^ region of brain lesions (Additional file 1: Fig. S2A–C). Meanwhile, a decrease in the numbers of SOX10^+^MYRF^+^ cells was observed in EAE mice compared with naїve mice (Fig. 1G, J). These observations suggested that activated microglia/macrophage blocks differentiation of OPCs in the lesion. Therefore, we hypothesized that activated microglia inhibit the expression of MYRF in OPCs, and in that way suppress their differentiation into OLGs. To test this, supernatant from SM or LPS-activated SM (SM-LPS) were added (50%) for 24 h to the OPCs. Compared with OPCs cultured in regular medium and OPCs cultured with SM supernatant, the expression of MYRF in OPCs incubated with SM-LPS supernatant was significantly decreased (Fig. 1K, L; Additional file 1: Fig. S3A–C). The above in vitro and in vivo results suggested that activated microglia decrease MYRF expression in OPCs via an interaction that does not require a direct cell–cell contact.

We then investigated the pathway that inhibits OPC differentiation through indirect cellular communication with activated microglia by OPCs cultured with SM- supernatant for 48 h, followed by immunofluorescence staining. The cultures with SM-LPS supernatant had increased percentages of PDGFRα^+^ OPCs (Fig. 1M, N), and reduced percentages of CNP^+^ OLGs (Fig. 1M, O). When comparing the degree of differentiation of OPCs, we found that the degree of differentiation of OPCs into OLGs was significantly lower in the SM-LPS Supernatant group than in the other groups (Fig. 1M, P). These results suggest a suppressive effect of microglia on OPC differentiation, through factors secreted by microglia. In addition, compared with healthy tissue of naïve mice, EAE mice had increased numbers of Iba1^+^ microglia/macrophage in the lesion (Fig. 1P,R), and decreased number of APC^+^ OLGs (Fig. 1P, Q). These in vitro and in vivo results indicate that microglia impede the differentiation of OPCs under inflammatory conditions.

### EXOs-LPS (exosomes in SM-LPS supernatant) inhibited OPCs differentiation

**Fig. 2:**
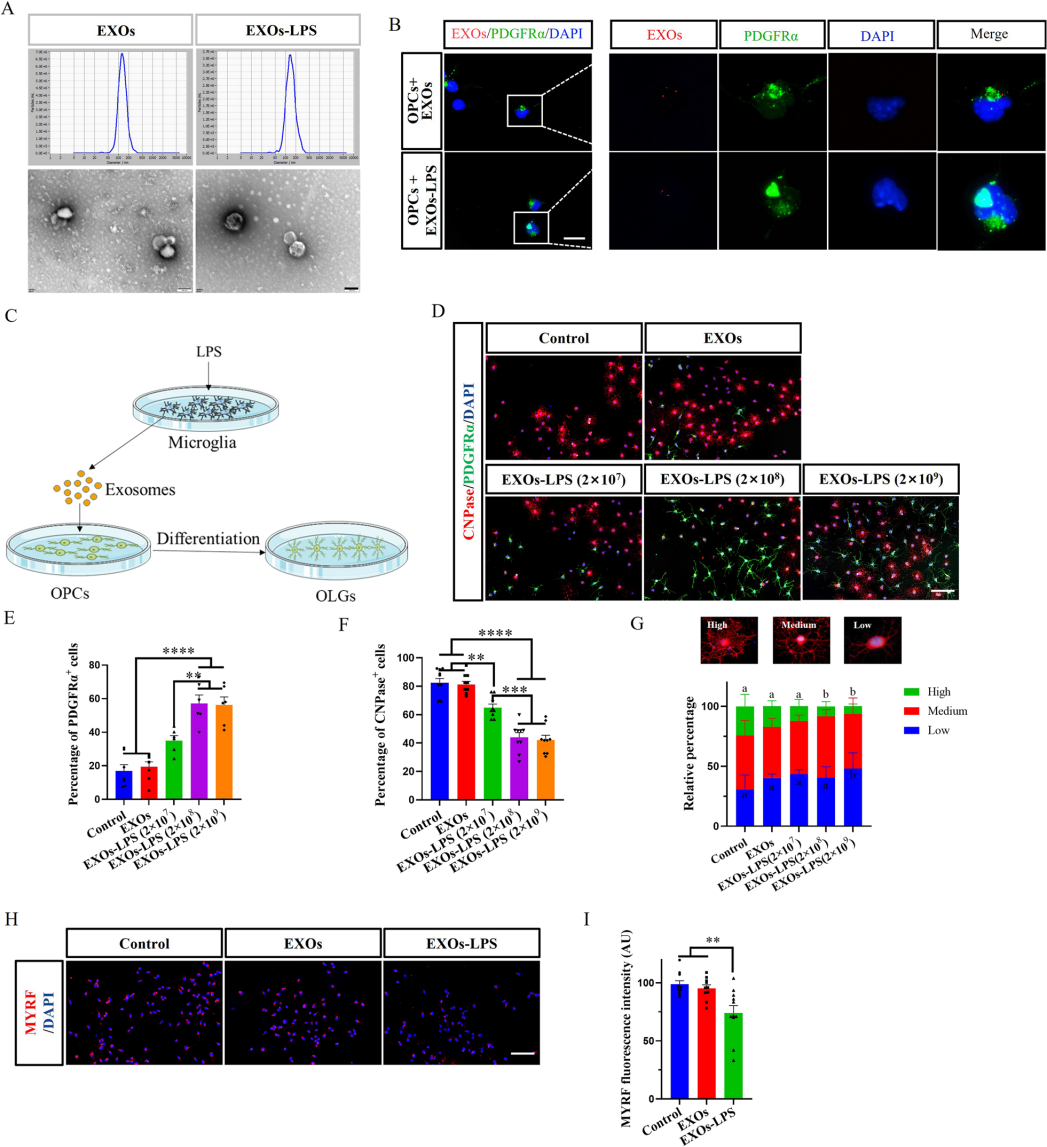
EXOs-LPS (exosomes in SM-LPS supernatant) inhibited OPCs differentiation. **A** NTA and TEM analyses to detect EXOs/EXOs-LPS particle size and apparent morphology. **B** Uptake of ExoGlow membrane labeling EXOs/EXOs-LPS by OPCs. **C** Schematic diagram of co-incubation of OPCs with EXOs/EXOs-LPS. **D** Immunofluorescence staining of OPCs differentiation after treatment with EXOs/EXOs-LPS, and **E**, **F** percentage of PDGFRα^+^ and CNPase^+^ cells. The dosage of EXOs was 2 × 10^8^ particles. **G** The relative percentage of three differentiation stages of OPCs in each experimental group. **H** Immunofluorescence staining to determine the expression intensity of MYRF after OPCs treatment with EXOs/EXOs-LPS, and **I** MYRF fluorescence intensity. Each data point represents the average of at least 4-6 regions of interest analyzed in each well, n ≥ 3 wells per group. All data are represented by mean ± SEM. One-way ANOVA was used to determine *P* values (**E**, **F**, **I**). **P < 0.01, ***P < 0.001, ****P < 0.0001. Two-way ANOVA was used to determine *P* values (**G**). Groups that do not share the same letter are significantly (P < 0.05). One representative of three independent experiments is shown. Scale bar = 100 nm (**A**), 20 μm (**B**), and 100 μm (**D**, **H**)

Our findings described in the preceding section indicated that activated microglia reduced OPC differentiation via a secretory factor. Such a factor could be delivered to OPCs within exosomes. To begin testing this possibility, we isolated exosomes from SM cells and tested their effect on OPC differentiation in vitro. First, we characterized isolated exosomes by Exoview (Additional file 1: Fig. S4A, B), NTA, and TEM (Fig. 2A) and verified the markers CD63 and CD9, morphology, and particle size. In order to test whether microglia could affect OPC differentiation by exosomes, we added EXOs (exosomes from SM supernatant) and EXOs-LPS (exosomes from SM-LPS supernatant) to OPC cultures. Immunofluorescence staining showed colocalization of OPCs and EXOs (Fig. 2B), which indicates that microglia could communicate with OPCs through exosomes. We then added EXOs/EXOs-LPS to OPCs under differentiating conditions to test whether EXOs-LPS will inhibit OPCs differentiation (Fig. 2C). As expected, OPC cultures with EXOs-LPS and had the most PDGFRα^+^ cells and the least CNPase^+^ cells in a dose-dependent manner and the degree of OPC differentiation was inhibited (Fig. 2D–G). Next, we tested whether EXOs-LPS impact the MYRF expression. OPCs incubated with EXOs-LPS had a lower MYRF expression as determined by immunofluorescence staining (Fig. 2H, I; Additional file 1: Fig. S3C, D). In conclusion, we demonstrate that exosomes could act as a communication medium between microglia and OPCs, and exosomes of activated microglia can inhibit the OPC differentiation.

### miR-615-5p was highly expressed in EXOs-LPS

**Fig. 3:**
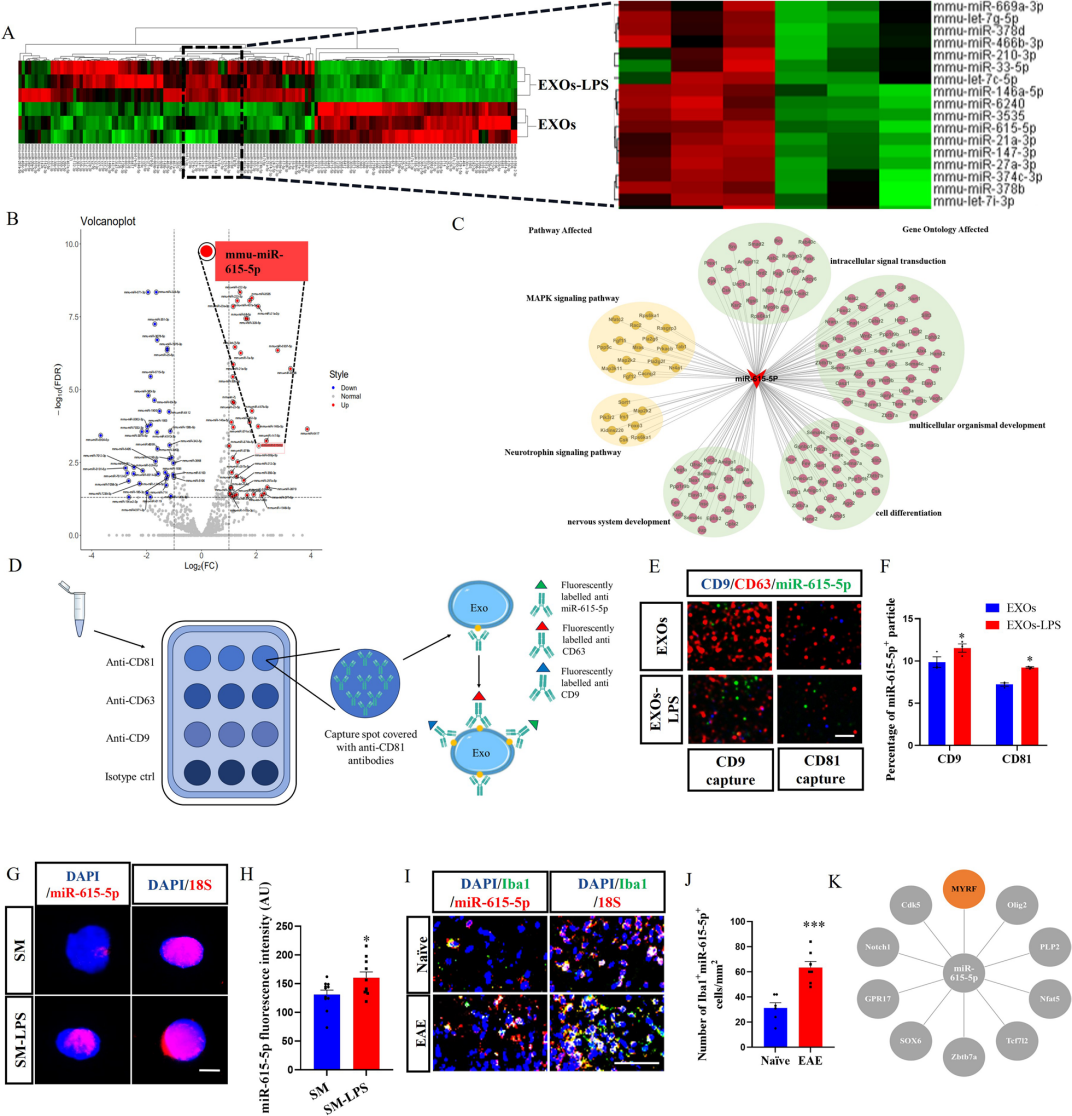
Exosome miRNA expression analysis and target gene prediction. **A** Gene expression heat maps and **B** volcano plots of miRNAs with significantly different expressions in EXOs/EXOs-LPS. **C** miR-615-5p target gene network expression map. **D** Schematic diagram of ExoView analysis. **E** ExoView detected the miR-615-5p of EXOs and EXOs-LPS by the specific probe and **F** the percentage of miR-615-5p^+^ particles. **G** FISH detected the expression of miR-615-5p in SM and SM-LPS, and **H** the miR-615-5p fluorescence intensity. **I** Immunofluorescence staining and FISH detected the expression of Iba1 and miR-615-5p in EAE/naïve spinal cords, and **J** the number of Iba1^+^miR-615-5p^+^ cells per mm^2^. **K** miRDB, TargetScan, and miRWalk online tools obtained miR-615-5p target genes related to OPCs differentiation. Sequencing data in 3 groups, each group n = 3 (**A**). Each data point represents the average of at least 4-6 regions of interest analyzed in each well, n ≥ 3 wells per group (**E**–**J**). All data are represented by mean ± SEM. *t*-tests were used to determine *P* values (**H**, **J**). Two-way ANOVA was used to determine *P* values (**F**). *P < 0.05, **P < 0.01. One representative of three independent experiments is shown. Scale bar = 5 μm (**E**), 20 μm (**G**) and 100 μm (**I**)

The above studies indicated that there was a clear cellular communication mode between OPCs and microglia through exosomes, which carried contents containing miRNA with unique biological activities, such as miRNA targeting and inhibiting the expression of its target genes in cells, thus achieving the function of information communication between cells [23, 24]. We therefore focused on exploring the mechanism of inhibition of OPC differentiation by EXOs-LPS via miRNA. Firstly, RNAs from EXOs and EXOs-LPS were sequenced, and compared to the miRNA database by Burrows–Wheeler Alignment (BWA) algorithm to obtain the relative expression levels of miRNAs. In the data analysis process, counts were standardized for more accurate results. We found significantly upregulated miR-615-5p expression as represented as a Heatmap and volcano map (Fig. 3A, B). GO analysis and Pathway enrichment showed that the potential target genes of miR-615-5p are involved in cell differentiation (Fig. 3C).

To verify the RNA-Seq results, Exoview, FISH, and qRT-PCR were performed to determine the expression level of miR-615-5p. Exoview confirmed that the proportion of miR-615-5p in EXOs-LPS was higher than in EXOs (Fig. 3D–F; Additional file 1: Fig. S3C, D). In addition, miR-615-5p immunoreactivity in the SM-LPS group was higher than that of the SM treated group by FISH (Fig. 3G, H). We then performed FISH and immunofluorescence staining in vivo and visually verified the expression of miR-615-5p in microglia of naїve and mice with EAE. The number of Iba1^+^miR-615-5p^+^ cells in the lumbar of the spinal cords of EAE mice was greater than in naïve mice (Fig. 3I, J). The number of PDGFRα/APC/GFAP/NeuN^+^miR-615-5p^+^ cells in the lumbar spinal cords of EAE mice and naïve mice showed no difference (Additional file 1: Fig. S5A–H). Moreover, the number of PDGFRα/APC/GFAP/NeuN^+^miR-615-5p^+^ cells was not as large as that of Iba1^+^miR-615-5p^+^ cells (Additional file 1: Fig. S5A–H). It is indicated that miR-615-5p is mainly expressed in the microglia but not in other neural cells. In addition, qRT-PCR analysis showed that miR-615-5p expression was substantially enhanced in the spinal cord and brain of EAE group compared with the naïve group (Additional file 1: Fig. S6A). SM and primary microglial cells were stimulated with LPS to detect miR-615-5p quantitatively. The expression of miR-615-5p was significantly upregulated in SM and primary microglia after LPS stimulation (Additional file 1: Fig. S6B, C). To determine endogenous miR-615-5p in OPCs, we isolated primary OPCs, extracted RNA after LPS stimulation, and conducted a quantitative analysis of miR-615-5p level. The results showed that the expression of miR-615-5p in OPCs was even lower than in non-stimulated microglia (Additional file 1: Fig. S6C). miR-615-5p immunoreactivity in the Raw264.7-LPS group was also higher than that of macrophage line Raw264.7 group by FISH (Additional file 1: Fig. S6D, E). The results implied that Iba1^+^ microglial or macrophages increased miR-615-5p levels after receiving inflammatory stimulation.

Although our data demonstrated increased expression of miR-615-5p in activated microglia, the underlying molecular mechanism whereby miR-615-5p could affect OPC was still unclear. Analysis via miRDB, TargetScan, and miRWalk online identified the top 10 target genes of miR-615-5p related to OPC differentiation (Fig. 3K), among which MYRF had the highest binding degree. Therefore, we speculated that miR-615-5p in EXOs-LPS targets MYRF and ultimately affects the differentiation of OPCs.

### Mechanisms of miR-615-5p in EXOs-LPS regulating OPCs differentiation

**Fig. 4:**
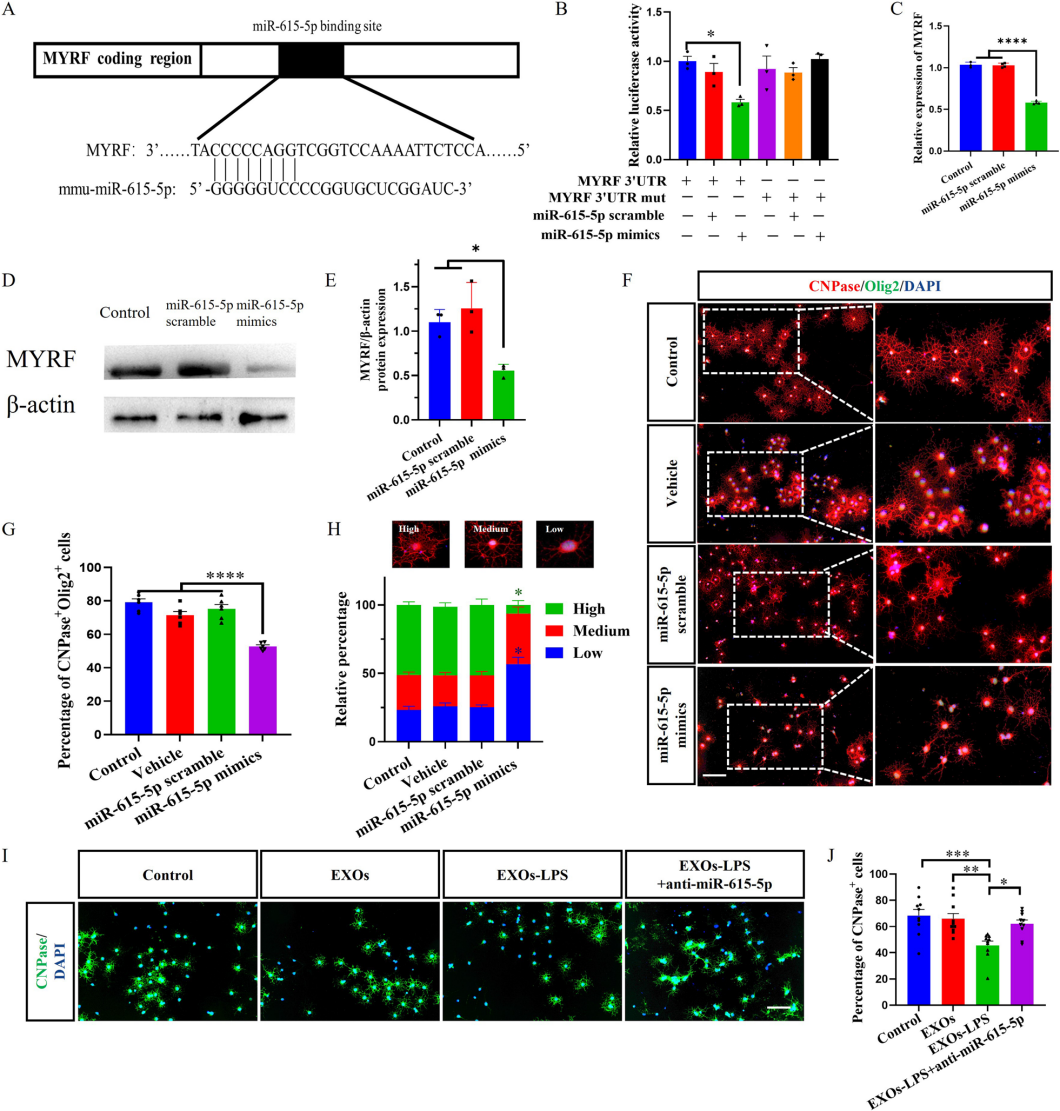
EXOs-LPS-derived miR-615-5p can target MYRF 3′UTR. **A** Schematic diagram of targeted binding sequence between miR-615-5p and MYRF 3′UTR. **B** Luciferase activity was tested by the one-GLo™ EX Luciferase Assay System in 293 T cells. **C** After OPCs transfection with miR-615-5p mimics, the expressions of MYRF and β-actin were detected by qRT-PCR. **D** After OPCs transfection with miR-615-5p mimics, the expressions of MYRF and β-actin were detected by WB, and **E** is the quantification of MYRF by Image J software. **F** CNPase and Olig2 were immunostained for the OPCs transfected with miR-615-5p mimics. The magnification of cells is shown on the right. **G** The percentage of CNPase^+^Olig2^+^ cell numbers divided by the total number of cells. **H** The relative percentage of three differentiation stages of OPCs in each experimental group. **I** Immunofluorescence staining to detect the expression of CNPase, and **J** the percentage of CNPase^+^ cells. Each data point represents the average of at least 4-6 regions of interest analyzed in each well, n ≥ 3 wells per group. All data are represented by mean ± SEM. T-tests were used to determine *P* values (**C**, **E**). One-way ANOVA was used to determine* P* values (**B**, **G**, **J**). *P < 0.05, **P < 0.01, ***P < 0.001. Two-way ANOVA was used to determine *P* values (**H**). One representative of three independent experiments is shown. Scale bar = 100 μm (**F**, **I**)

miR-615-5p was highly enriched in EXOs-LPS, and specify name of the sites predicted that miR-615-5p binds to MYRF. We therefore focused on the mechanism whereby miR-615-5p regulates MYRF expression and its effect on OPCs differentiation. We used luciferase assay to verify that miR-615-5p binds MYRF. The binding diagram of MYRF 3′UTR and miR-615-5p is shown in Fig. 4A. According to the luciferase report assay, miR-615-5p mimics resulted in the decreased activity of MYRF, indicating the direct binding of miR-615-5p to MYRF (Fig. 4B). These results suggested that miR-615-5p directly binds to 3′UTR of MYRF.

We then transfected OPCs with synthetic miR-615-5p scramble and miR-615-5p mimics, cultured them in a proliferation medium for 24 h, and determined MYRF expression by qRT-PCR and WB. The MYRF mRNA and protein levels in OPCs transfected with miR-615-5p mimics were significantly reduced compared with the OPCs cultured with medium alone and transfected with miR-615-5p scramble group (Fig. 4C–E), indicating that miR-615-5p regulates the expression of MYRF.

Next, OPCs were transfected with synthetic miR-615-5p mimics or scrambled control, and immunofluorescence staining was performed after 2 days of culture in the differentiation medium. The percentage of CNPase^+^Olig2^+^ cells in the miR-615-5p mimics transfection group was significantly reduced compared with control groups (Fig. 4F, G). When the degrees of OPC differentiation were compared, we found that OPCs in the miR-615-5p mimics transfection group were notably less differentiated into OLGs than in other groups (Fig. 4F, H). These data indicated that miR-615-5p reduces differentiation of OPCs to OLGs and their maturation.

To further verify the role of miR-615-5p in EXOs-LPS, SM cells were treated with anti-miR-615-5p mimics and LPS, and then EXOs-LPS + anti-miR-615-5p was isolated. Finally, 2 × 10^8^ particles of EXOs, EXOs-LPS, or EXOs-LPS + anti-miR-615-5p were added to OPCs cultures to determine the effect of antagonizing miR-615-5p in EXOs-LPS on OPC development. In contrast to EXOs-LPS, EXOs-LPS + anti-miR-615-5p did not block OPCs differentiation (Fig. 4I, J), suggesting that EXOs-LPS-derived miR-615-5p inhibited the differentiation of OPCs.

We also investigated the effects of miR-615-5p in EXOs-LPS on mRNA levels of other target genes for miR-615-5p by qRT-PCR. The relative expression of miR-615-5p target genes, such as Plp2, Tcf7l2, Cdk5, and Sox6 were not significantly reduced after adding the supernatant of activated SM cells to OPC cultures (Additional file 1: Fig. S7A–D). These data suggested that the inhibitory effects of miR-615-5p on OPC differentiation are not likely achieved through silencing of these genes.

### Antagonizing miR-615-5p in microglia alleviated EAE development

**Fig. 5:**
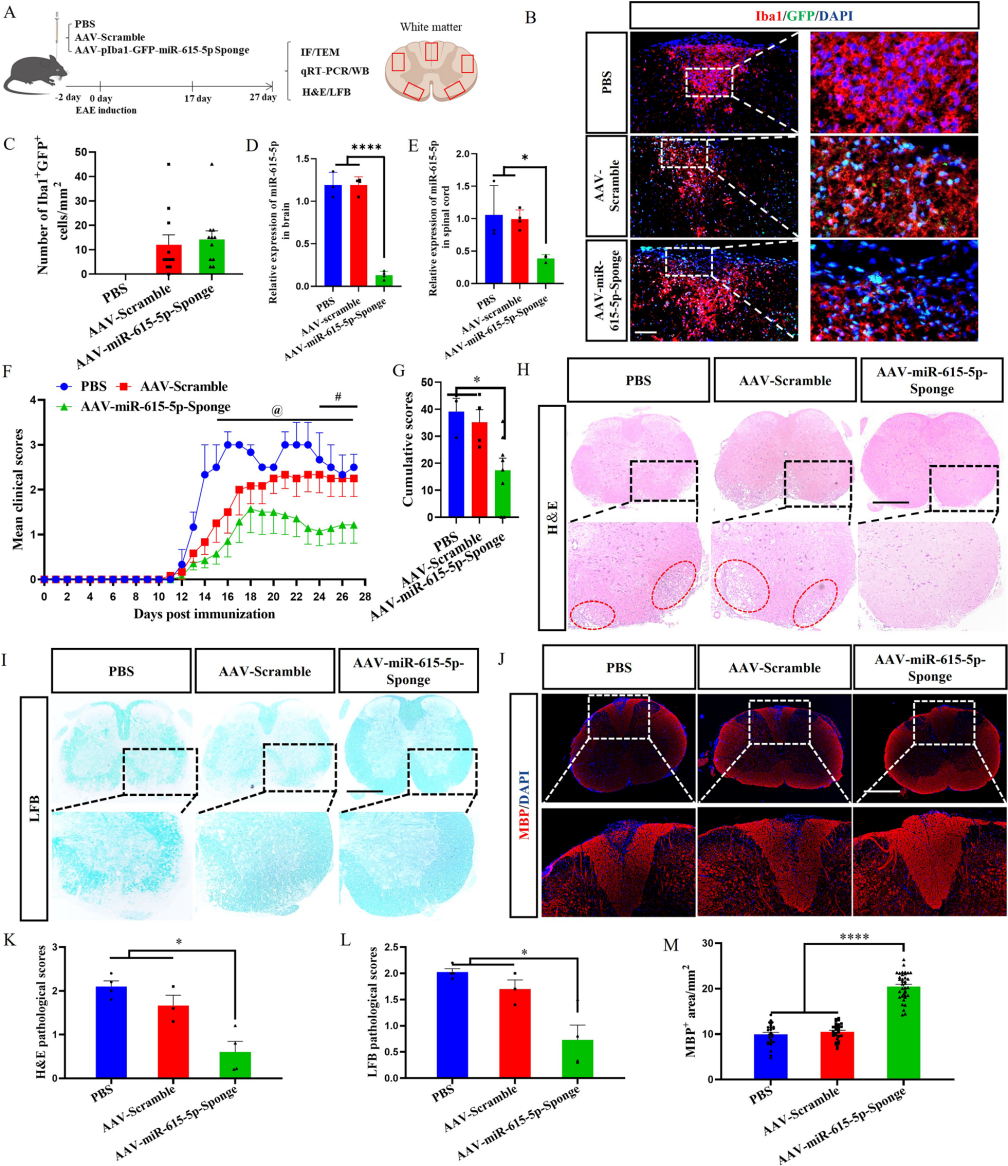
Antagonizing miR-615-5p in microglia significantly alleviated EAE development. **A** Mice were divided into three groups: PBS group, AAV-Scramble group, and AAV-miR-615-5p-Sponge group. Two days before immunization, AAVs were injected into mice via the lateral ventricles. After 27 days of immunization, spinal cords, and brains were harvested, and HE, LFB, IF, TEM, qRT-PCR, and WB were performed. **B** Immunofluorescence staining of Iba1 and GFP and local magnification in each group is shown on the right, and **C** the number of Iba1^+^GFP^+^ cells per mm^2^. **D**, **E** qRT-PCR analysis of miR-615-5p in mouse brain and spinal cords. **F** Clinical score of EAE mice. The @ symbol represents the comparison between the PBS group and the AAV-miR-615-5p-Sponge group. The # symbol represents the AAV-Scramble group compared with the AAV-miR-615-5p-Sponge group. **G** The cumulative scores were calculated from day 10 to day 27 p.i. **H** Spinal cords were assayed for H&E staining, **I** LFB staining, and **J** MBP fluorescence staining. The lower rows of (**K**–**M**) showed the locally enlarged images of H&E, LFB, and MBP staining in each group. The red circle represents inflammatory cell infiltration in (**H**). **K**, **L** Statistical results of H&E and LFB pathological scores. **M** MBP^+^ area per mm^2^. Each data point represents the average of 4–6 regions of interest analyzed per section from at least 3 sections per animal with n ≥ 3 mice per group. Spinal cord samples analyzed in the white matter lesion area (**B**, **H**, **I**, **J**). Typical representative figures show the dorsal cord lesion area of the spinal cord (**B**, **J**). All data are represented by mean ± SEM. One-way ANOVA was used to determine *P* values (**C**, **D**, **E**, **G**, **K**, **M**, **L**). Two-way ANOVA was used to determine *P* values (**E**). *@P* < 0.05 (day 15-27)*, #P* < 0.05 (day 24–27), *P < 0.05, **P < 0.01, ****P < 0.0001. One representative of three independent experiments is shown. Scale bar = 100 μm (**B**) and 500 μm (**G**–**I**)

We then tested whether blocking miR-615-5p in microglia can restore remyelination in EAE. The experimental design is shown in Fig. 5A. Iba1 staining was performed on spinal cords of mice at day 27 p.i. Given that AAV carries the GFP gene regulated by the Iba1 promoter, colocalization of GFP^+^ and Iba1^+^ cells were observed in the AAV-miR-615-5p-Sponge group and the AAV-Scramble group but not in the PBS group (Fig. 5B, C). The level of miR-615-5p expression in AAV-miR-615-5p-Sponge group was lower than that in the other two groups as determined by qRT-PCR (Fig. 5D, E). These results confirmed that AAV virus was injected successfully and exerted the biological function in microglia of EAE mice.

The EAE mice were scored blindly from the day of immunization to assess the therapeutic effect of injected AAVs. The clinical scores of the AAV-miR-615-5p-Sponge-treated group were consistently lower than that of the PBS- and AAV-Scramble-treated groups (Fig. 5F), and their cumulative scores were significantly reduced as well (Fig. 5G). The number of inflammatory infiltrating cells (as detected by H&E staining; Fig. 5H, K) and extent of demyelination (as detected by LFB staining; Fig. 5I, L) in the AAV-miR-615-5p-Sponge-treated mice were significantly reduced compared with those in the PBS and AAV-Scramble groups. In contrast, immunofluorescence staining showed that the MBP fluorescence intensity in the white matter area of the spinal cords in the AAV-miR-615-5p-Sponge group was notably higher than that in the PBS and the AAV-Scramble group (Fig. 5J, M). In AAV-miR-615-5p-Sponge-treated mice, the number of astrocyte marker GFAP^+^ and Anti-Amyloid Precursor Protein^+^ (APP^+^) cells was remarkably lower than that of PBS/AAV-Scramble-treated mice (Additional file 1: Fig. S8A–C). These data show that inhibiting the expression of miR-615-5p could alleviate clinical disease in EAE and reduce demyelination.

### Antagonizing miR-615-5p increased the expression of the MYRF gene and alleviated demyelination in EAE mice.

**Fig. 6:**
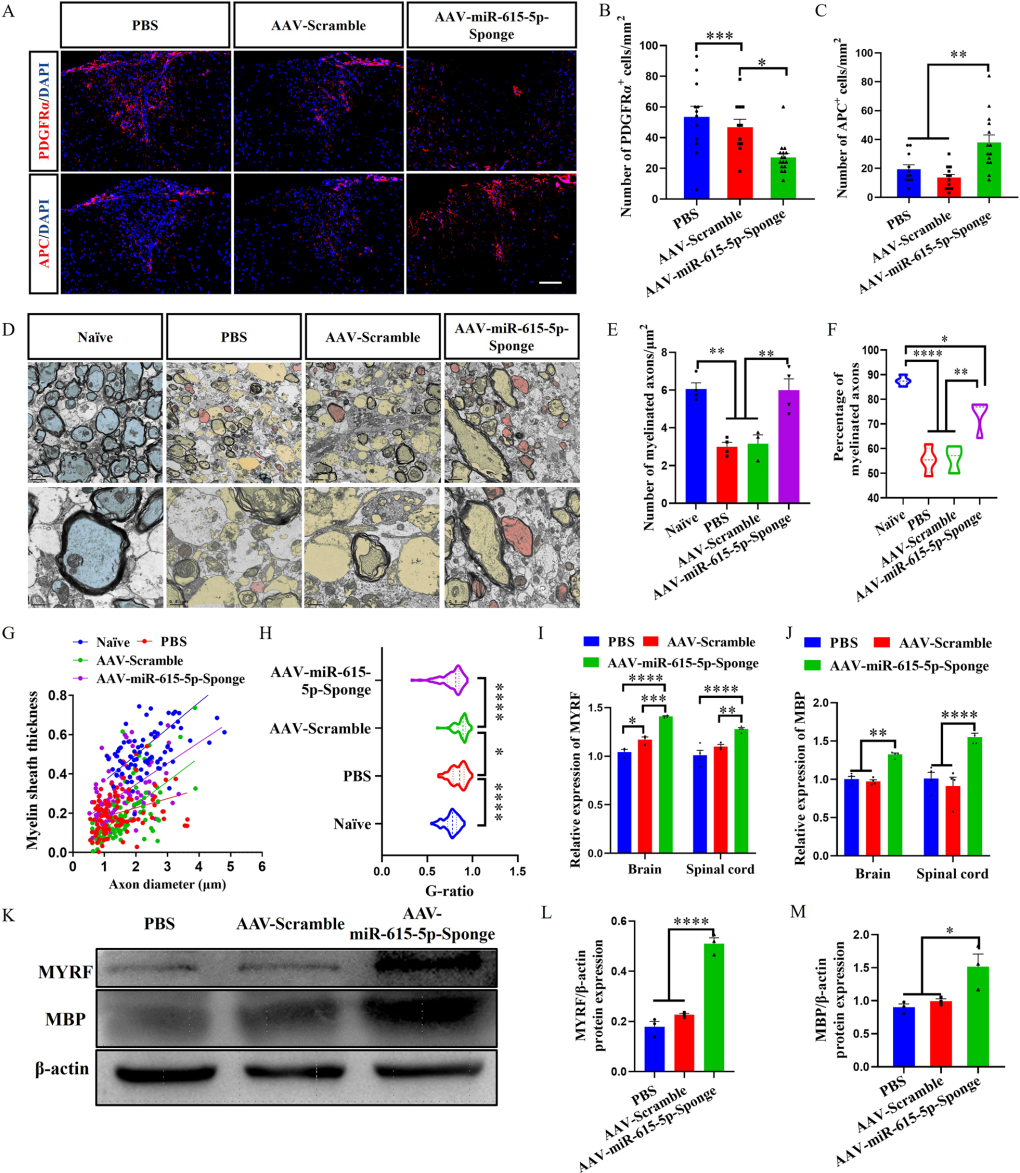
Antagonizing miR-615-5p can alleviate demyelination in EAE mice. **A** Immunolabeling of PDGFRα and APC in the spinal cords, and **B**, **C** the number of PDGFRα^+^ and APC^+^ cells per mm^2^. **D** TEM images of the spinal cords in different experimental groups. Magnified views of partial myelin sheaths are shown below. Red represents regenerated myelin, yellow represents demyelinated sheath, and blue represents normal myelin. **E** Number of remyelinated axons. **F** Percentage of myelinated axons to total axons. **G** Scatter plot of myelin sheath thickness. **H** Quantification of the G-ratios (axon diameter/fiber diameter) of myelinated fibers. **I**, **J** The expression of MYRF and MBP mRNA was detected by qRT-PCR in the brain and spinal cords. **K** After antagonizing miR-615-5p, the expressions of MYRF, MBP, and β-actin in the spinal cords were detected by WB. **L**, **M** Image J software quantification statistical results. Each data point represents the average of 4–6 regions of interest analyzed per section from at least 3 sections per animal with *n* ≥ 3 mice per group. Spinal cord samples analyzed in the white matter lesion area (**A**, **D**). Typical representative figures show the dorsal cord lesion are of the spinal cord(A). All data are represented by mean ± SEM. One-way ANOVA was used to determine *P* values (**B**, **C**, **E**–**H**, **L**, **M**). Two-way ANOVA was used to determine *P* values (**I**, **J**). *P < 0.05, **P < 0.01, ***P < 0.001, ****P < 0.0001. One representative of three independent experiments is shown. Scale bar = 100 μm (**A**) and 2 μm (**D**)

The spinal cords of EAE mice described in the preceding section were harvested for PDGFRα and APC immunofluorescence staining to characterize the proportion of OPCs and OLs, respectively. Compared with the PBS and AAV-Scramble groups, the number of PDGFRα^+^ OPCs in the AAV-miR-615-5p-Sponge group was reduced (Fig. 6A, B), with increased number of APC^+^ OLGs (Fig. 6A, C). These results indicated that neutralizing miR-615-5p in the microglia of EAE mice could promote OPCs differentiation. TEM images showed that the myelin sheaths in the lumbar enlargement of naïve mice were dense and thick, while the myelin sheaths in the PBS and AAV-Scramble groups were thin and loose (Fig. 6D, G). Furthermore, the myelin sheaths of mice in the AAV-miR-615-5p-Sponge group were thicker than those of the PBS and AAV-Scramble groups (Fig. 6D), with increased numbers (Fig. 6E) and percentages (Fig. 6F) of remyelinated axons. The G-ratio values of the naїve and miR-615-5p-Sponge groups were lower than those of the PBS and AAV-Scramble groups (Fig. 6H). These results indicated that the AAV-miR-615-5p-Sponge as a therapeutic reagent exerts a function of alleviating demyelination.

qRT-PCR and WB results showed that the expression of MYRF mRNA and protein in the AAV-miR-615-5p-Sponge group was significantly higher than that of the PBS and AAV-Scramble groups (Fig. 6I, K, L). As a transcription factor, MYRF enhance the expression of the downstream gene MBP. We thus detected the expression level of MBP. Compared with those in the PBS and the AAV-Scramble group, MBP mRNA and protein levels in the AAV-miR-615-5p-Sponge group were significantly greater (Fig. 6J, K, M). Thus, antagonizing miR-615-5p significantly increased the expression of MYRF protein and its downstream target gene MBP and achieved the therapeutic effect of alleviating demyelination.

### Antagonizing miR-615-5p in microglia alleviated demyelination in CPZ model

**Fig. 7:**
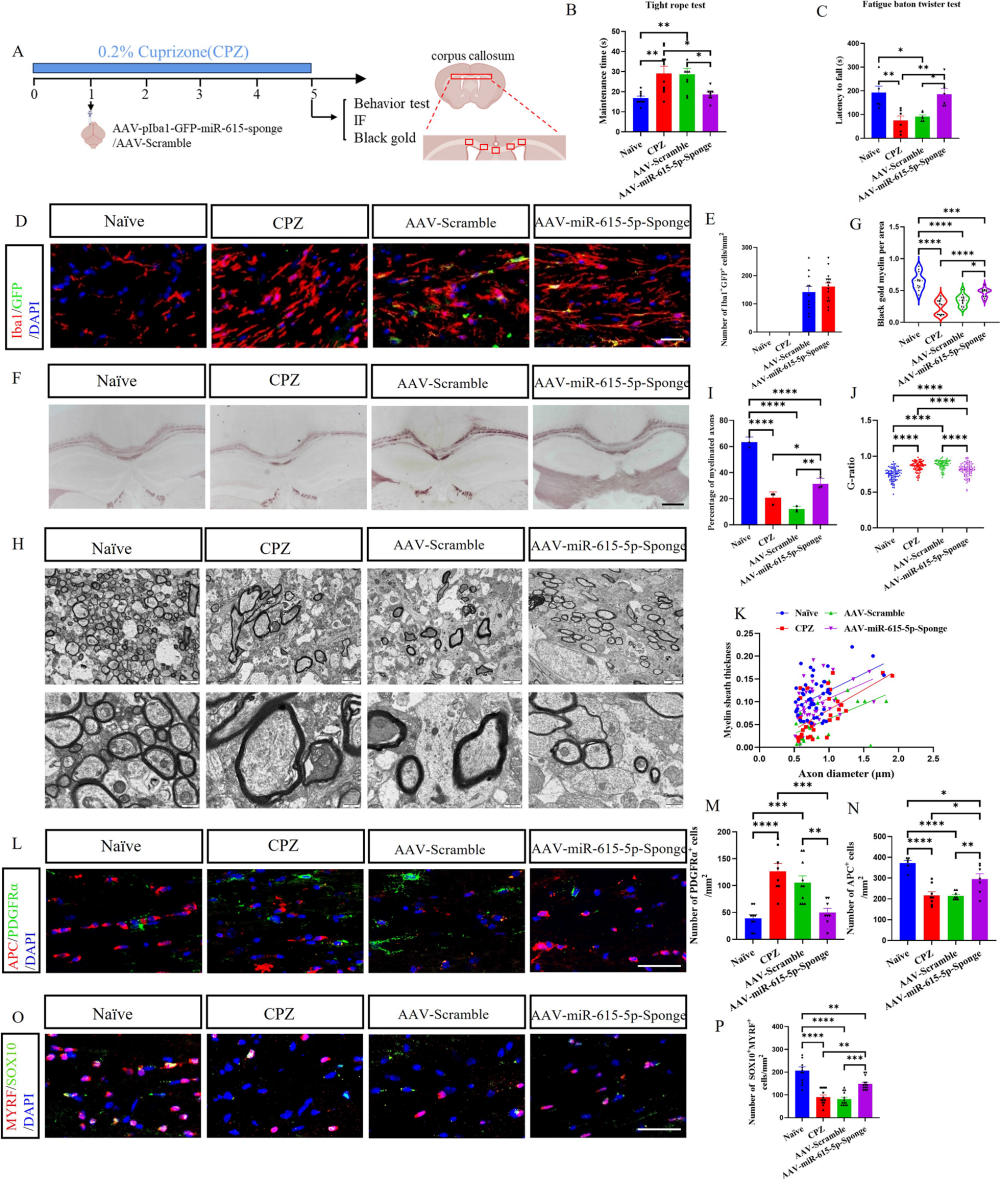
Antagonizing miR-615-5p in microglia significantly alleviated demyelination in the CPZ model. **A** Mice were divided into four groups: Naïve group, CPZ group, AAV-Scramble group, and AAV-miR-615-5p-Sponge group. One week after feeding 0.2% CPZ diet, AAVs were injected into mice via the lateral ventricles. After 5 weeks of feeding 0.2% CPZ diet, the behavioral test was performed, and brains were harvested for black gold and IF testing. **B**, **C** The motor coordinative function was assessed by tight rope test and fatigue baton twister test. **D** Immunofluorescence staining of Iba1 and GFP in each group is shown, and **E** the number of Iba1^+^GFP^+^ cells per mm^2^. **F** Black gold staining of myelinated axons and **G** Black gold myelin per area. **H** TEM images of the brains in different experimental groups. **I** Percentage of myelinated axons to total axons. **J** Quantifying the G-ratios (axon diameter/fiber diameter) of myelinated fibers. **K** Scatter plots of myelin sheath thickness. **L** Immunolabeling of PDGFRα and APC in the Brains, and **M**, **N** the number of PDGFRα^+^ and APC^+^ cells per mm^2^. **O** Immunolabeling of MYRF and SOX10 in the Brains, and **P** the number of MYRF^+^ SOX10^+^ cells per mm^2^. Each data point represents the average of 4–6 regions of interest analyzed per section from at least 3 sections per animal with n ≥ 3 mice per group. Sites of lesions in the CC region analyzed in mouse brain samples. One-way ANOVA was used to determine *P* values (**B**, **C**, **E**, **G**, **I**, **J**, **K**, **M**, **N**, **P**). *P < 0.05, **P < 0.01, ***P < 0.001, ****P < 0.0001. One representative of three independent experiments is shown. Scale bar = 50 μm (**D**, **L**, **O**) and 5 μm (**F**)

It has been demonstrated that the activation of microglia plays a crucial role in the CPZ-induced demyelination process [2]. Therefore, we tested whether blocking miR-615-5p in microglia can ameliorate remyelination in the drug-induced demyelination model. The experimental design is shown in Fig. 7A. Tight rope test and fatigue baton twister test were used to assess the motor coordinative function of mice, which is affected by CPZ-induced demyelination. A more severe locomotor impairment condition was observed in mice in the CPZ and AAV-Scramble groups compared to the naïve group, but this condition was alleviated in the AAV-miR-615-5p-Sponge group (Fig. 7B, C). Iba1 staining of mice brains at 4 weeks post-AAV administration. Given that AAV carries the GFP gene regulated by the Iba1 promoter, colocalization of GFP^+^ and Iba1^+^ cells were observed in the AAV-miR-615-5p-Sponge group and the AAV-Scramble group but not in the naïve group and CPZ group (Fig. 7D, E). This result confirmed that the AAV virus was injected successfully and exerted the biological function in the microglia of CPZ mice.

Demyelination was assessed by black gold staining of CPZ mice brains to observe the corpus callosum (CC). Compared with the naïve group, the CPZ and AAV-Scramble group were severely demyelinated, but demyelination was significantly reduced in the AAV-miR-615-5p-Sponge group (Fig. 7F, G). TEM images showed that the myelin sheaths in the brain of naïve mice were dense and thick, while the myelin sheaths in the CPZ and AAV-Scramble groups were thin and loose (Fig. 7H). Furthermore, the myelin sheaths of mice in the AAV-miR-615-5p-Sponge group were thicker than those of the CPZ and AAV-Scramble groups (Fig. 7K), with increased percentages (Fig. 7I) of myelinated axons. The G-ratio values of the naїve and miR-615-5p-Sponge groups were lower than those of the CPZ and AAV-Scramble groups (Fig. 7J). These results suggest that AAV-miR-615-5p-Sponge, as a therapeutic reagent, protects myelin and prevents myelin sheath loss. The mouse CC was taken for PDGFRα and APC immunofluorescence staining, and the number of PDGFRα^+^ OPCs in the AAV-miR-615-5p-Sponge group decreased the number of APC^+^ OLGs increased compared with that in the CPZ group and the AAV-Scramble group (Fig. 7L–N). The results suggest that neutralizing miR-615-5p in the microglia of CPZ mice promotes the differentiation of OPCs. MYRF and SOX10 immunofluorescence staining was next performed to characterize the expression of MYRF in the OLs, and MYRF^+^SOX10^+^ cells were significantly reduced in the CPZ group and AAV-Scramble group compared to the naïve group, but the number of MYRF^+^SOX10^+^ cells was again increased in the AAV-miR-615-5p-Sponge group (Fig. 7O, P). Thus, antagonizing miR-615-5p significantly increased MYRF protein expression and achieved a therapeutic effect of inhibiting demyelination.

## Discussion

**Fig. 8:**
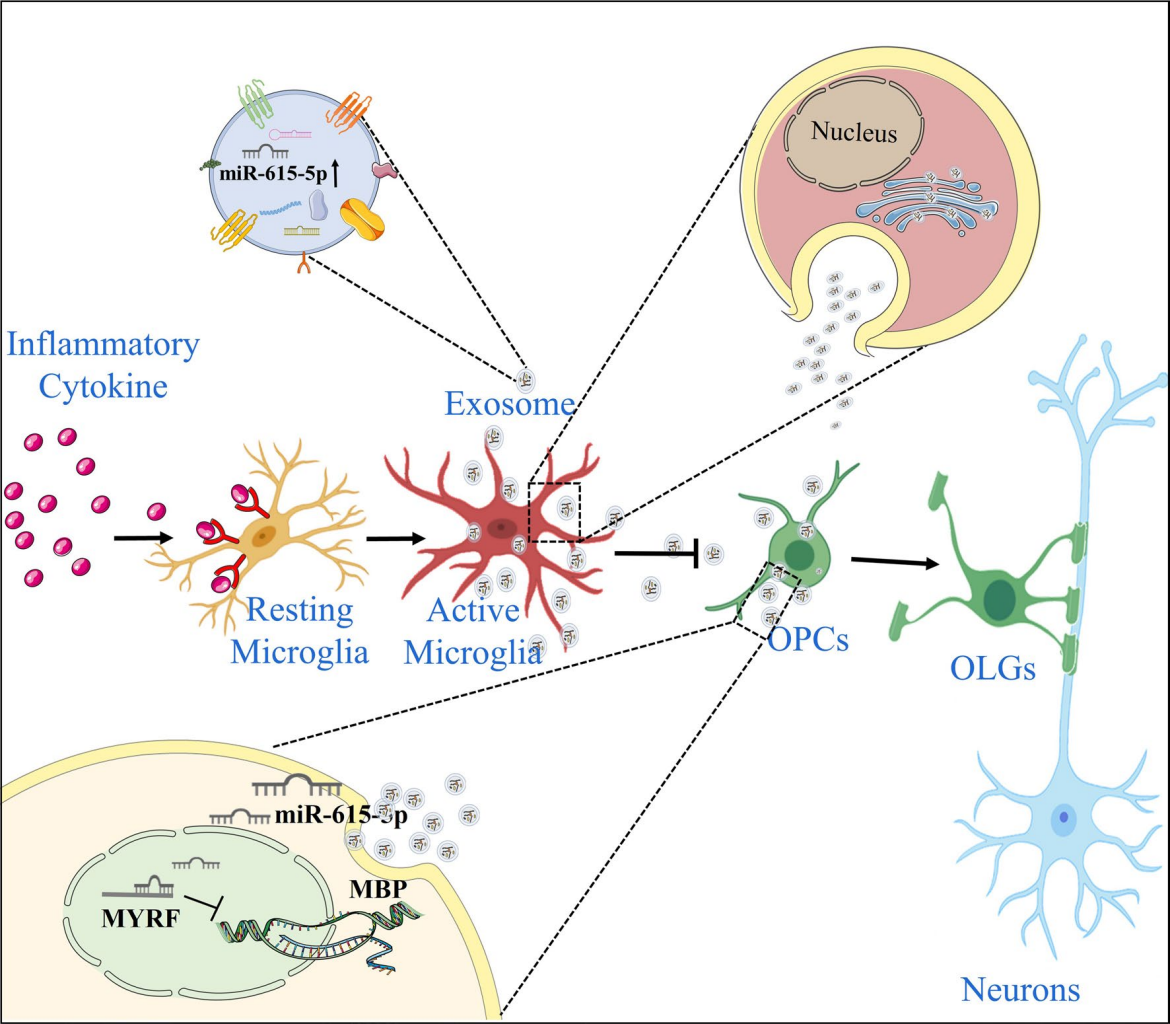
Diagram of mechanism. In an inflammatory environment, resting microglia transform into activated microglia. The exosomes released by activated microglia can serve as their cellular communication with OPCs. In addition, miR-615-5p was expressed highly in activated microglia-derived exosomes, which silenced MYRF and ultimately led to the obstruction of OPCs differentiation

In the present study, we demonstrate that activated microglia/infiltrated macrophages secreted exosomes with a high level of miR-615-5p, which silenced MYRF and affected the expression of its downstream gene MBP, eventually leading to the arrest of OPCs differentiation (Fig. 8).

In multiple CNS diseases, many inflammation-related cells and mediators form an inflammatory microenvironment, which is a common pathological feature that drives the development of these diseases [25]. For example, microglia functions are unbalanced in traumatic spinal cord injury, constituting a pro-inflammatory microenvironment. It has been speculated that immunomodulatory microglia can secrete exosomes to regulate neuronal damage, while the specific regulatory mechanism is unknown [26]. Indirect communication that does not require cell contact is a type of cell communication, in which cells regulate behavior and transmit signals by secreting cytokines, growth factors, and exosomes [27, 28]. Exosomes contain proteins and nucleic acids that interact with and modify local and distant cellular targets, thus play a major role in cell communication [29]. In the CNS microenvironment, miRNAs in microglia-derived exosomes are critical for neurogenesis in depression. Fan et al. demonstrated that miR-146a-5p derived from microglia shuttled into neurons, inhibited CDKL5 expression by binding to KLF4, enhanced p-STAT3 levels, and thus reduced neurogenesis and induced depression-like behavior [30]. There is increasing evidence that exosomes released by microglia play a significant role in myelination. These exosomes act as communication carriers by carrying miRNA. Li et al.’s research has shown that M2 microglia can communicate with OPCs via exosomes, promoting white matter repair. The interaction between M2 microglia and OPCs leads to increased OPC proliferation, survival, and maturation, resulting in improved white matter repair. By utilizing exosomes derived from M2 microglia, it may be possible to enhance OPC survival and development, thereby improving outcomes for conditions such as stroke and demyelinating diseases. This discovery paves the way for the development of novel therapies in the treatment of these conditions [31]. These findings suggest that exosomes play an essential role in intercellular regulation as a new communication mode. In this study, we extracted RNA from exosomes isolated from activated microglia and found that miR-615 was highly enriched in these exosomes. The existence of miR-615 in a single exosome was further confirmed by NanoView combined with a probe. These results prove that exosomes can effectively deliver genetic materials to target cells and regulate biological activity.

Here, we identified miR-615-5p as a potential therapeutic approach to promote OPCs differentiation. Previous research has discovered that miR-615 can interact with several target genes, such as Sox5, IGF2, and EGFR, which play crucial roles in cell proliferation and differentiation [32–34]. Inhibiting miR-615 can release the negative regulation on these target genes, increasing expression levels and promoting cell growth. However, all of the above conclusions are found in tumor cells. Therefore, the inhibition of miR-615 is not expected to achieve a similar trend in OPCs. In addition to the known target genes, other yet undiscovered potential targets may exist by inhibiting miR-615. Further research and experiments could unveil new target genes closely associated with myelin sheath generation. These novel target genes may regulate the process of myelin sheath formation, providing new targets and strategies for developing therapeutic interventions.

Our results suggested that exosomes released by microglia in a CNS inflammatory microenvironment result in the differentiation blockade of OPCs (Fig. 2). This might be due to the inhibition of intrinsic OPCs differentiation-related transcription factors, e.g., MYRF, by miRNAs carried by activated microglia via released exosomes. A series of scenarios have also been indicated, in which OPCs differentiation could be affected by pro-inflammatory cytokines such as IFN-γ, TNF-α accumulation in the lesion [35]. Further, OPCs show apparent heterogeneity in the inflammatory microenvironment, i.e., a part of OPCs express MHCII and may possess some functions of immune cells [5]. The effects of inflammatory cytokines mentioned above on the differentiation of OPCs are obvious in EAE, an autoimmune disease model. However, in certain toxic-induced demyelination models such as cuprizone, microglial activation is the major pathogenesis while CNS inflammation is minor. Indeed, we have shown that the expression level of miR-615 in microglia was significantly higher than that in OPCs. In vitro, when OPCs were stimulated with LPS, the expression of miR-615 was not significantly affected, supporting the view that miR-615 was directly transferred from exosomes to OPCs. In addition, we demonstrated the ability of OPCs to ingest exosomes derived from microglia. Exosomes have recently emerged as paracrine mediators contributing to intra- and inter-tissue communication. A recent study also revealed the critical role of miR-330 transferred from stem cells from human exfoliated deciduous teeth-derived exosomes in regulating microglia, providing further evidence suggests that exosomes emerge as paracrine mediators contributing to intra- and inter-tissue communication [36]. In particular, miRNAs in exosomes can regulate communication between different cells, thereby regulating gene expression and target cell function.

It is worth noting that the current research field on miR-615 is mainly reflected in the expression of brain tissue during embryonic development, and little information is known about the biological function of this miRNA in the CNS, especially in pathological conditions. miR-615 also appears to be linked to some extent to neurodegenerative diseases such as Huntington's disease and Alzheimer's disease [37]. We have for the first identified miR-615, a crucial component of exosomes released from activated microglia, as a candidate miRNA silencing MYRF, thus blocking myelination/remyelination. This novel finding was further confirmed by our in vivo study showing that administration of AAV-pIba1-miR-615-sponge to the EAE mice alleviates the disease development and promotes remyelination, which represents a promising therapeutic approach to demyelinating diseases such as MS.

### Supplementary Information

**Additional file 1:** **Figure S1.** The map of plasmid. **A** MYRF 3′UTR-Luciferase vector. **B** Mut MYRF 3′UTR-Luciferase vector. **Figure S2.** The expression of MBP and Iba1 in MS Lesion. **A** Immunofluorescence staining of MBP and Iba1 in brain of NAWM and MS lesion and **B**, **C** MBP fluorescence intensity and the number of Ibal^+^ cells per field. Scale bar = 100 μm. All data are represented by mean ± SEM (*n* = 5, each group). One-way ANOVA was used to determine *P* values (**B**, **C**). ****p < 0.0001. **Figure S3.** The expression of PDGFRα and MYRF. **A** After co-incubation of OPCs with supernatant, the expression of PDGFRα and MYRF and **B** the percentage of PDGFRα^+^MYRF^+^ cells were detected by immunofluorescence staining. **C** Immunofluorescence staining to determine the PDGFRα and MYRF expression intensity after OPCs treatment with EXOs/EXOs-LPS, and **D** the percentage of PDGFRα + MYRF + cells. One-way ANOVA was used to determine *P* values. *P < 0.05, **P < 0.01, ****P < 0.0001. Scale bar = 50 μm. **Figure S4.** Expression of miR-615-5p in EXOs/EXOs-LPS. **A**, **B** After exosomes were captured by CD81/CD9 chip, miR-615-5p was detected in EXOs/EXOs-LPS by Exoview. In addition, surface markers CD63 and CD9 of EXOs/EXOs-LPS were detected by Exoview. **C**, **D** ExoView detected the average colocalization percent of EXOs or EXOs-LPS. **Figure S5.** The co-expression of miR-615-5p and PDGFRα, APC, GFAP and NeuN. **A** Immunofluorescence staining and FISH detected the expression of PDGFRα and miR-615-5p in EAE/naïve spinal cords, and **B** the number of PDGFRα^+^miR-615-5p^+^ cells per field. **C** Immunofluorescence staining and FISH detected the expression of APC and miR-615-5p in EAE/naïve spinal cords, and **D** the number of APC ^+^miR-615-5p^+^ cells per field. **E** Immunofluorescence staining and FISH detected the expression of GFAP and miR-615-5p in EAE/naïve spinal cords, and **F** the number of GFAP^+^miR-615-5p^+^ cells per field. **G** Immunofluorescence staining and FISH detected the expression of NeuN and miR-615-5p in EAE/naïve spinal cords, and **H** the number of NeuN ^+^miR-615-5p^+^ cells per field. Scale bar = 50 μm. All data are represented by mean ± SEM (*n* = 5, each group). One-way ANOVA was used to determine *P* values. **Figure S6.** Expression of miR-615-5p. **A** The expression of miR-615-5p was detected by qRT-PCR in the brain and spinal cords of naïve/EAE mice. **B** The expression of miR-615-5p was detected by qRT-PCR in SM and SM-LPS. **C** The expression of miR-615-5p was detected by qRT-PCR in microglia, activated microglia, OPCs, and activated OPCs. **D** Raw264.7 was stimulated with 100 ng/ml LPS. FISH detected the expression of miR-615-5p in Raw264.7 and Raw264.7-LPS, and **E** the miR-615-5p fluorescence intensity. All data are mean ± SEM. *t*-tests were used to determine *P* values (**B**, **E**). One-way ANOVA was used to determine *P* values (**C**). Two-way ANOVA was used to determine p values (**A**). *P < 0.05, **P < 0.01, ***P < 0.001, ****P < 0.0001. One representative of three independent experiments is shown. **Figure S7.** Expression of miR-615-5p target genes. **A**–**F** After OPCs were incubated with SM-derived supernatant/SM-LPS-derived supernatant, qRT-PCR was used to detect the other target genes of miR-615-5p, including Plp2, Tcf7l2, Cdk5, Sox6. T-test was used to determine *P* values. **P < 0.01, ****P < 0.0001. One representative of three independent experiments is shown. **Figure S8.** Antagonistic miR-615-5p inhibited the expression of GFAP and APP. **A** Immunofluorescence staining of GFAP and APP in spinal cords of naïve and EAE mice, and **B**, **C** the number of GFAP^+^ cells and APP^+^ cells per field. Scale bar = 100 μm. All data are represented by mean ± SEM (*n* = 10, each group). One-way ANOVA was used to determine *P* values (**B**, **C**). ****p < 0.0001. One representative of three independent experiments is shown. **Table S1.** Primers for PCR. **Table S2.** Primers for qRT-PCR. **Table S3.** miR-615-5p target gene network expression table.

## Data Availability

The data that support the findings of this study are available from the corresponding author upon reasonable request.

## Abbreviations

CNS: Central nervous system
MS: Multiple sclerosis
OPCs: Oligodendrocyte precursor cells
OLGs: Oligodendrocytes
EAE: Experimental autoimmune encephalomyelitis
MYRF: Myelin regulator factor
miRNA: MicroRNA
PTX: Pertussis toxin
CFA: Complete Freund's adjuvant
SM cells: Mouse microglia SIM-A9
LPS: Lipopolysaccharide
EXOs: Exosomes in SM supernatant
EXOs-LPS: Exosomes in SM-LPS supernatant
NTA: Nanoparticle tracking analysis
TEM: Transmission electron microscopy
FISH: Fluorescence in situ hybridization
WT: Wild-type
MUT: Mutant
