# Supplementary material for: Microglia-derived exosomes modulate myelin regeneration via miR-615-5p/MYRF axis
Source: J Neuroinflammation. 2024 Jan 22;21:29. doi: 10.1186/s12974-024-03019-5 (PMC10801965; doi:10.1186/s12974-024-03019-5)
Supplement: Supplementary file 1 — Additional file 1: Figure S1. The map of plasmid. A MYRF 3′UTR-Luciferase vector. B Mut MYRF 3′UTR-Luciferase vector. Figure S2. The expression of MBP and Iba1 in MS Lesion. A Immunofluorescence staining of MBP and Iba1 in brain of NAWM and MS lesion and B, C MBP fluorescence intensity and the number of Ibal+ cells per field. Scale bar = 100 μm. All data are represented by mean ± SEM (n = 5, each group). One-way ANOVA was used to determine P values (B, C). ****p < 0.0001. Figure S3. The expression of PDGFRα and MYRF. A After co-incubation of OPCs with supernatant, the expression of PDGFRα and MYRF and B the percentage of PDGFRα+MYRF+ cells were detected by immunofluorescence staining. C Immunofluorescence staining to determine the PDGFRα and MYRF expression intensity after OPCs treatment with EXOs/EXOs-LPS, and D the percentage of PDGFRα + MYRF + cells. One-way ANOVA was used to determine P values. *P < 0.05, **P < 0.01, ****P < 0.0001. Scale bar = 50 μm. Figure S4. Expression of miR-615-5p in EXOs/EXOs-LPS. A, B After exosomes were captured by CD81/CD9 chip, miR-615-5p was detected in EXOs/EXOs-LPS by Exoview. In addition, surface markers CD63 and CD9 of EXOs/EXOs-LPS were detected by Exoview. C, D ExoView detected the average colocalization percent of EXOs or EXOs-LPS. Figure S5. The co-expression of miR-615-5p and PDGFRα, APC, GFAP and NeuN. A Immunofluorescence staining and FISH detected the expression of PDGFRα and miR-615-5p in EAE/naïve spinal cords, and B the number of PDGFRα+miR-615-5p+ cells per field. C Immunofluorescence staining and FISH detected the expression of APC and miR-615-5p in EAE/naïve spinal cords, and D the number of APC +miR-615-5p+ cells per field. E Immunofluorescence staining and FISH detected the expression of GFAP and miR-615-5p in EAE/naïve spinal cords, and F the number of GFAP+miR-615-5p+ cells per field. G Immunofluorescence staining and FISH detected the expression of NeuN and miR-615-5p in EAE/naïve spinal cords, and H the number [file 12974_2024_3019_MOESM1_ESM.docx]

**
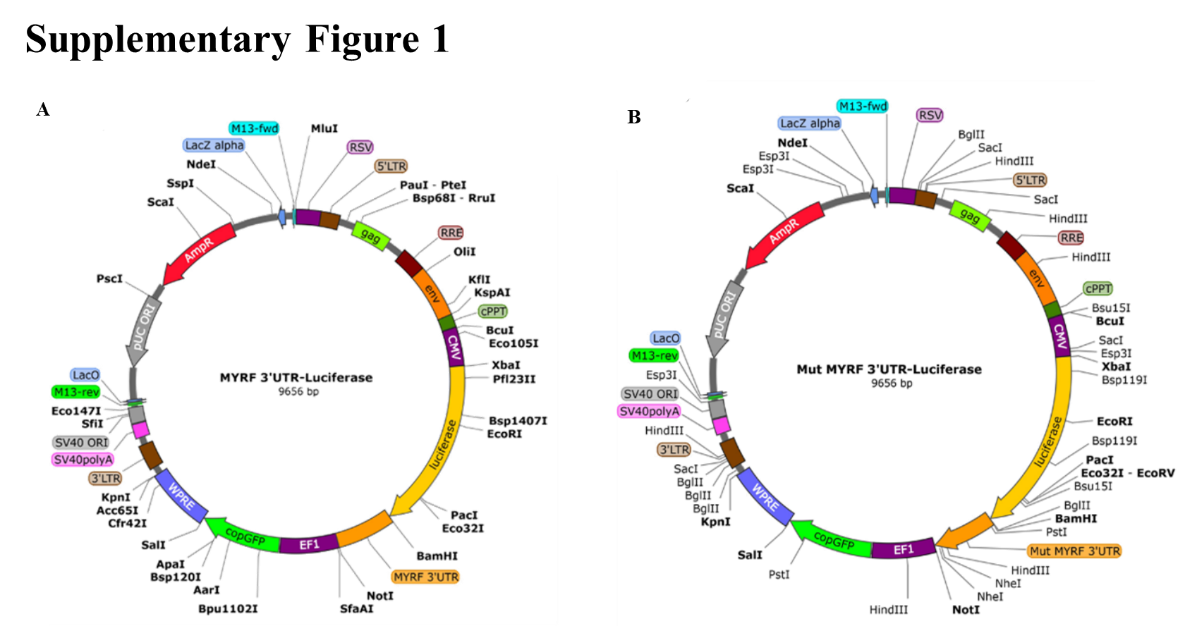
**

**Supplementary Figure 1. The map of plasmid.**

1. MYRF 3’UTR-Luciferase vector. **(B)** Mut MYRF 3’UTR-Luciferase vector.


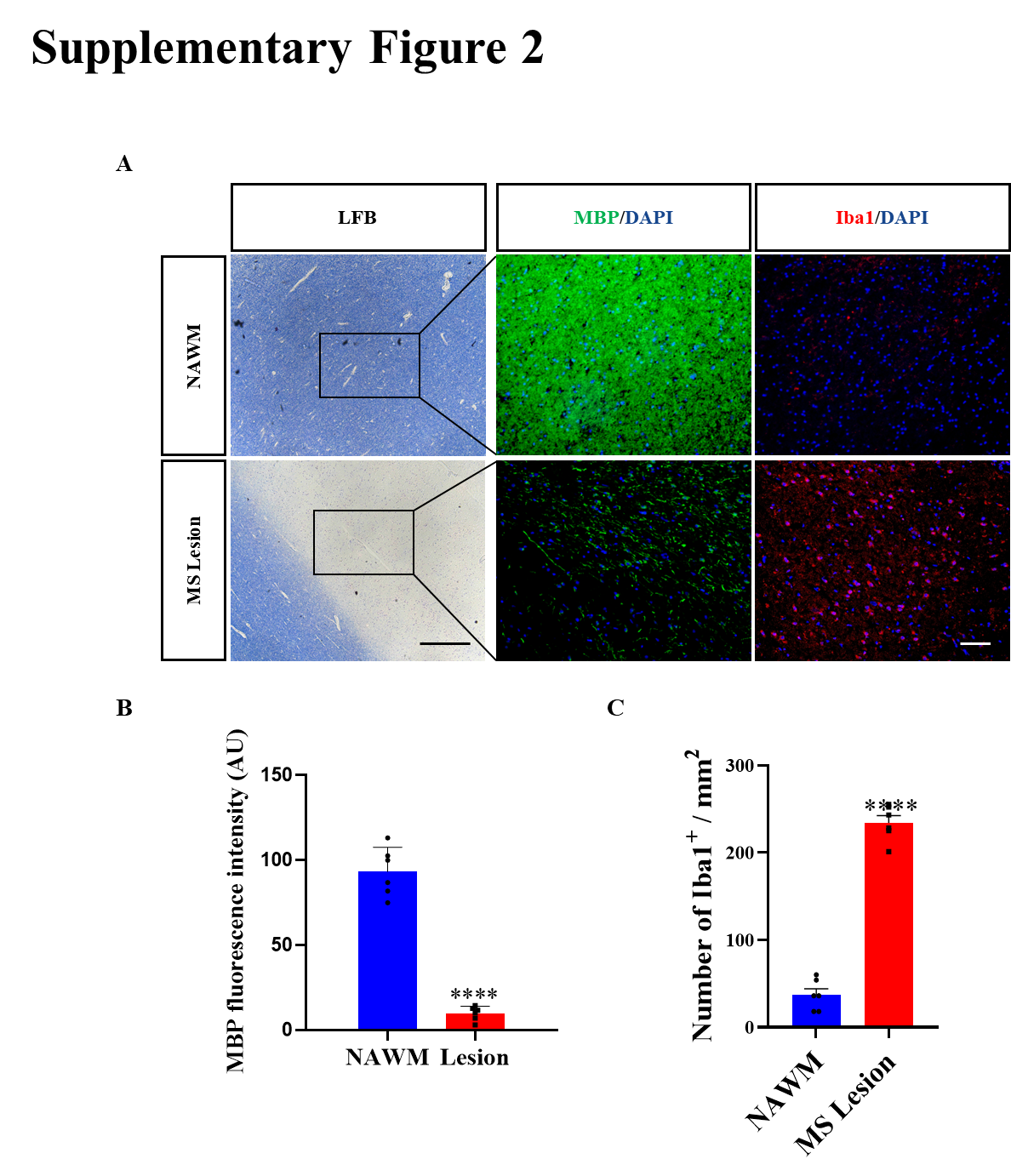


**Supplementary Figure 2. The expression of MBP and Iba1 in MS Lesion.**

**(A)** Immunofluorescence staining of MBP and Iba1 in brain of NAWM and MS lesion and **(B, C)** MBP fluorescence intensity and the number of Ibal^+^ cells per field. Scale bar = 100 μm. All data are represented by mean ± SEM (n = 5, each group). One-way ANOVA was used to determine *P* values (B, C). ****p＜0.0001.

**
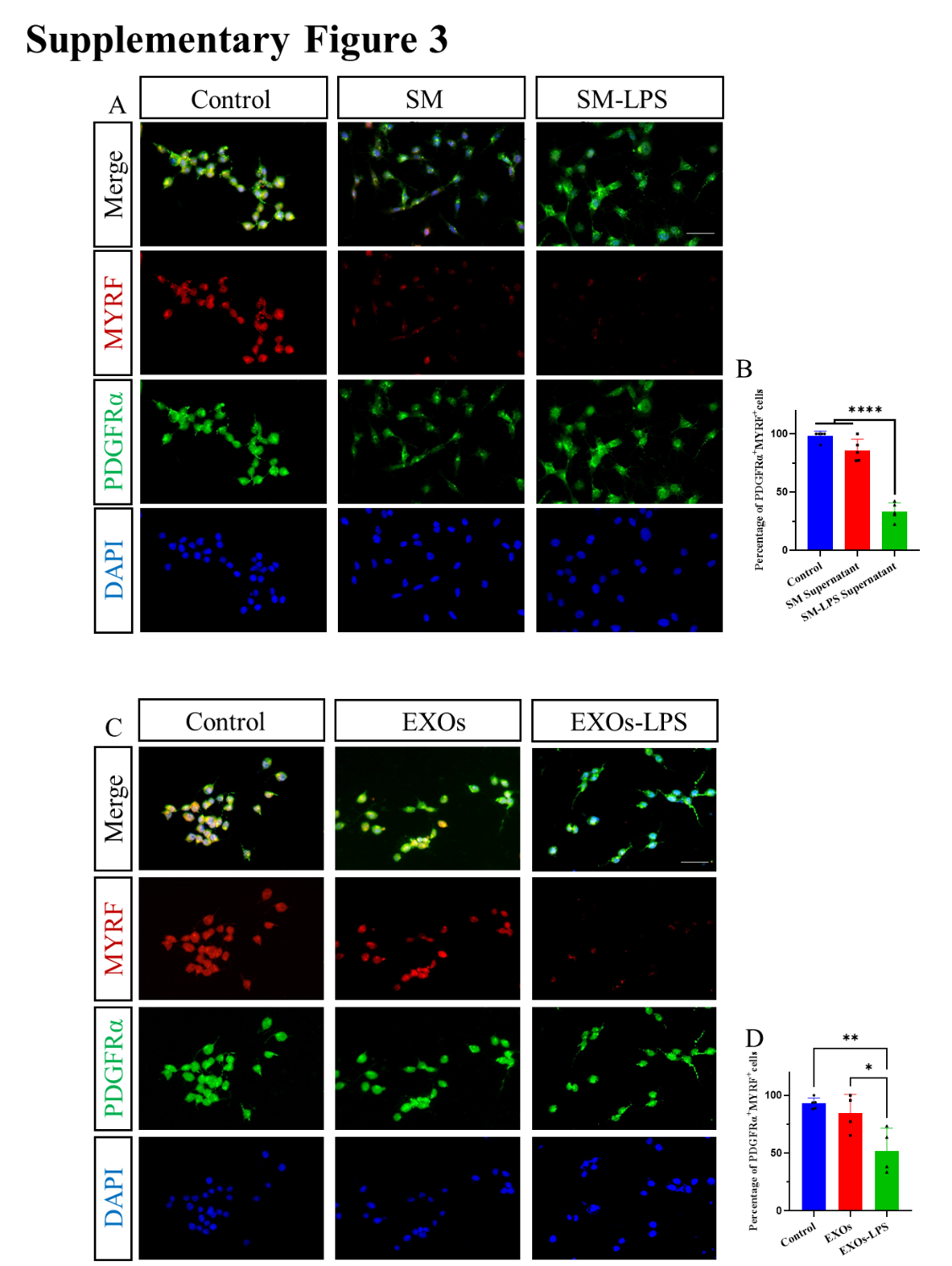
**

**Supplementary Figure 3. The expression of PDGFRα and MYRF**

**(A)** After co-incubation of OPCs with supernatant, the expression of PDGFRα and MYRF and **(B)** the percentage of PDGFRα^+^MYRF^+^ cells were detected by immunofluorescence staining. **(C)** Immunofluorescence staining to determine the PDGFRα and MYRF expression intensity after OPCs treatment with EXOs/EXOs-LPS, and **(D)** the percentage of PDGFRα+MYRF+ cells. One-way ANOVA was used to determine P values. *P < 0.05, **P < 0.01， *****P* ＜0.0001. Scale bar = 50 μm.

**
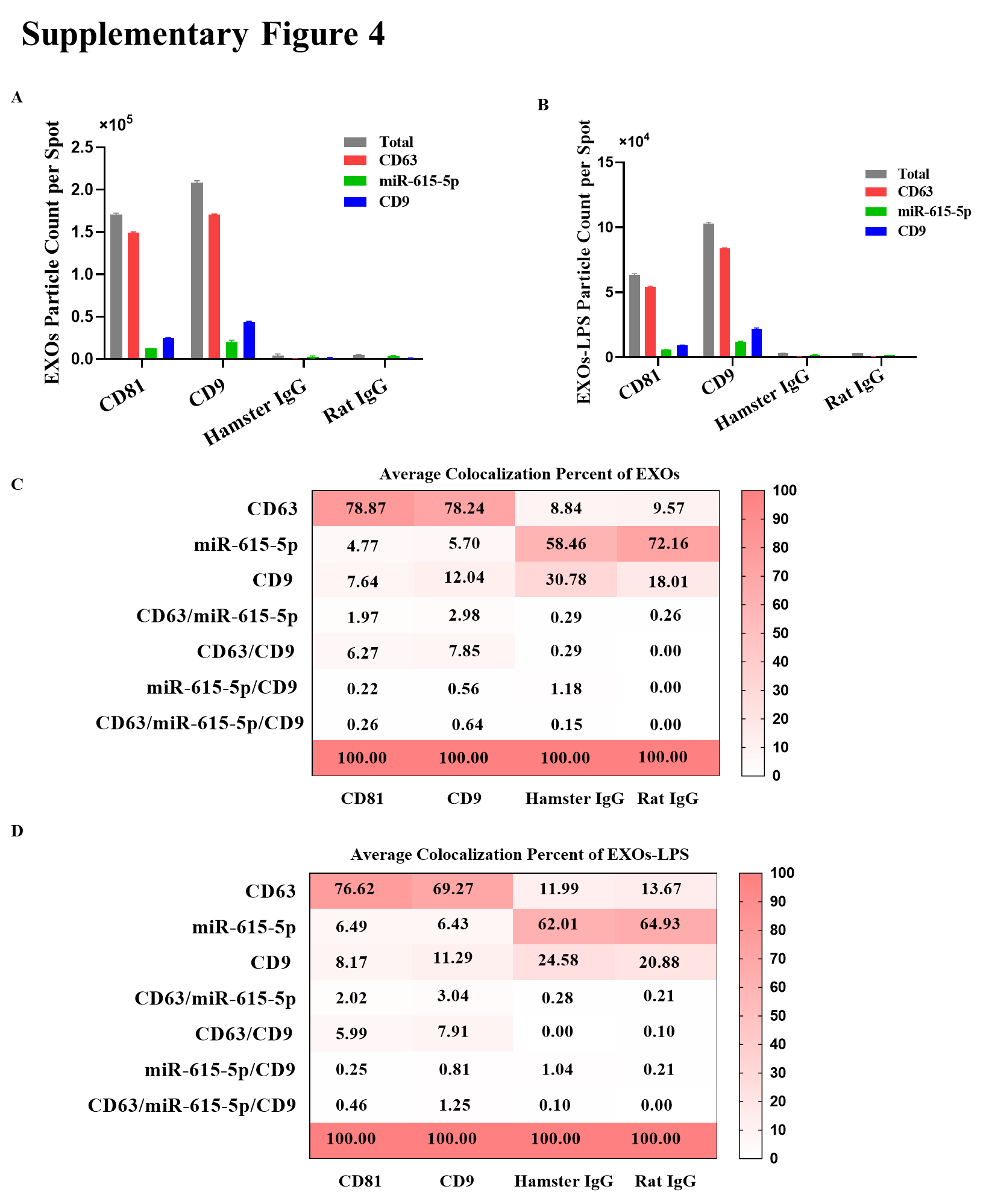
**

**Supplementary Figure 4. Expression of miR-615-5p in EXOs/EXOs-LPS.**

**(A, B)** After exosomes were captured by CD81/CD9 chip, miR-615-5p was detected in EXOs/EXOs-LPS by Exoview. In addition, surface markers CD63 and CD9 of EXOs/EXOs-LPS were detected by Exoview. **(C, D)** ExoView detected the average colocalization percent of EXOs or EXOs-LPS.

**
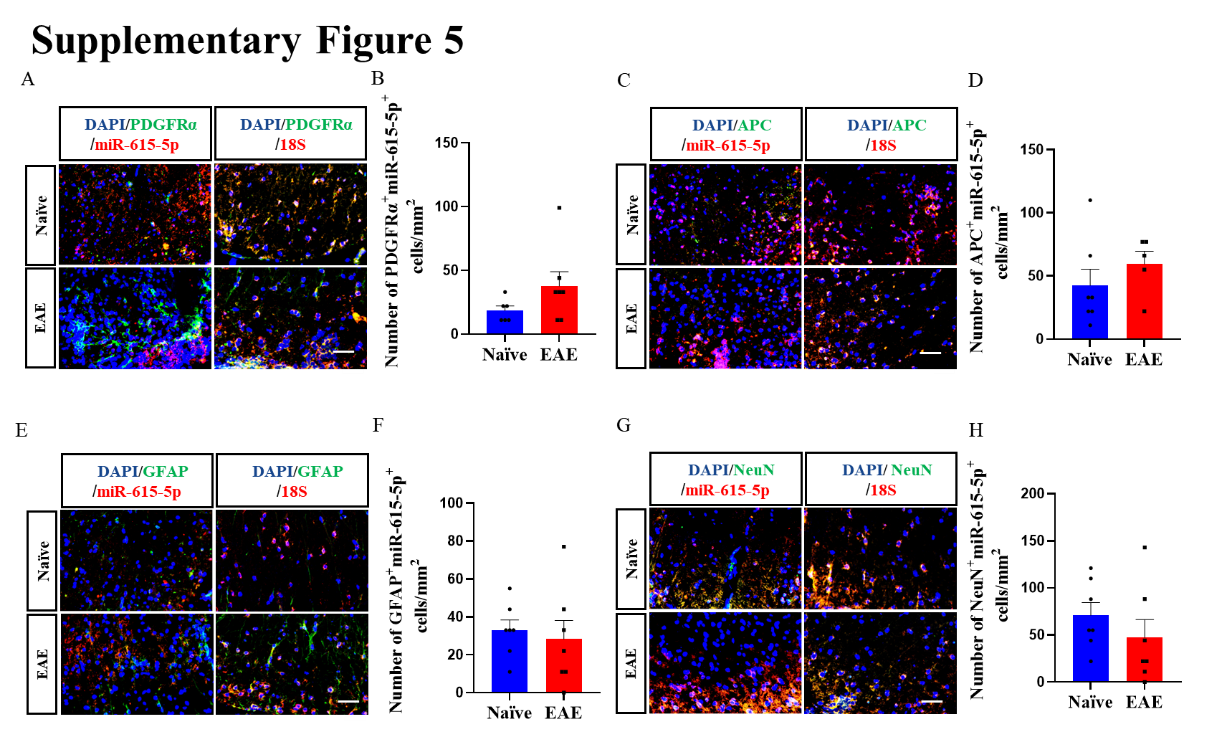
**

**Supplementary Figure 5. The co-expression of** **miR-615-5p and** **PDGFRα, APC, GFAP and NeuN.**

**(A)** Immunofluorescence staining and FISH detected the expression of PDGFRα and miR-615-5p in EAE/naïve spinal cords, and **(B)** the number of PDGFRα^+^miR-615-5p^+^ cells per field. **(C)** Immunofluorescence staining and FISH detected the expression of APC and miR-615-5p in EAE/naïve spinal cords, and **(D)** the number of APC ^+^miR-615-5p^+^ cells per field. **(E)** Immunofluorescence staining and FISH detected the expression of GFAP and miR-615-5p in EAE/naïve spinal cords, and **(F)** the number of GFAP^+^miR-615-5p^+^ cells per field. **(G)** Immunofluorescence staining and FISH detected the expression of NeuN and miR-615-5p in EAE/naïve spinal cords, and **(H)** the number of NeuN ^+^miR-615-5p^+^ cells per field. Scale bar = 50 μm. All data are represented by mean ± SEM (n = 5, each group). One-way ANOVA was used to determine *P* values.

**
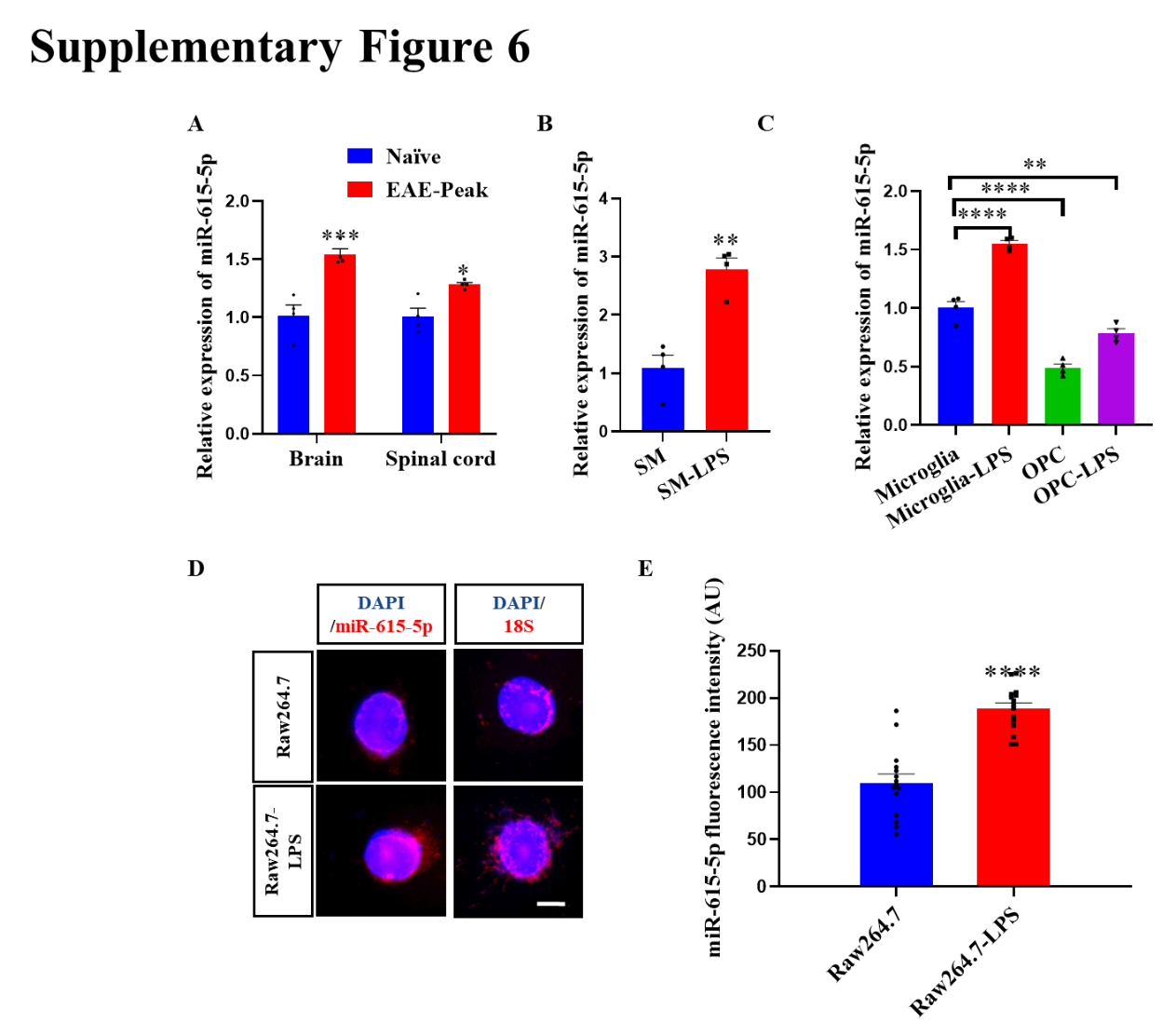
**

**Supplementary Figure 6. Expression of miR-615-5p.**

**(A)** The expression of miR-615-5p was detected by qRT-PCR in the brain and spinal cords of naïve/EAE mice. **(B)** The expression of miR-615-5p was detected by qRT-PCR in SM and SM-LPS. **(C)** The expression of miR-615-5p was detected by qRT-PCR in microglia, activated microglia, OPCs, and activated OPCs. **(D)** Raw264.7 was stimulated with 100 ng/ml LPS. FISH detected the expression of miR-615-5p in Raw264.7 and Raw264.7-LPS, and **(E)** the miR-615-5p fluorescence intensity. All data are mean ± SEM. T-tests were used to determine *P* values (B, E). One-way ANOVA was used to determine *P* values (C). Two-way ANOVA was used to determine p values (A). **P* < 0.05, ** *P* < 0.01, ***P < 0.001, **** *P*＜0.0001. One representative of three independent experiments is shown.

**
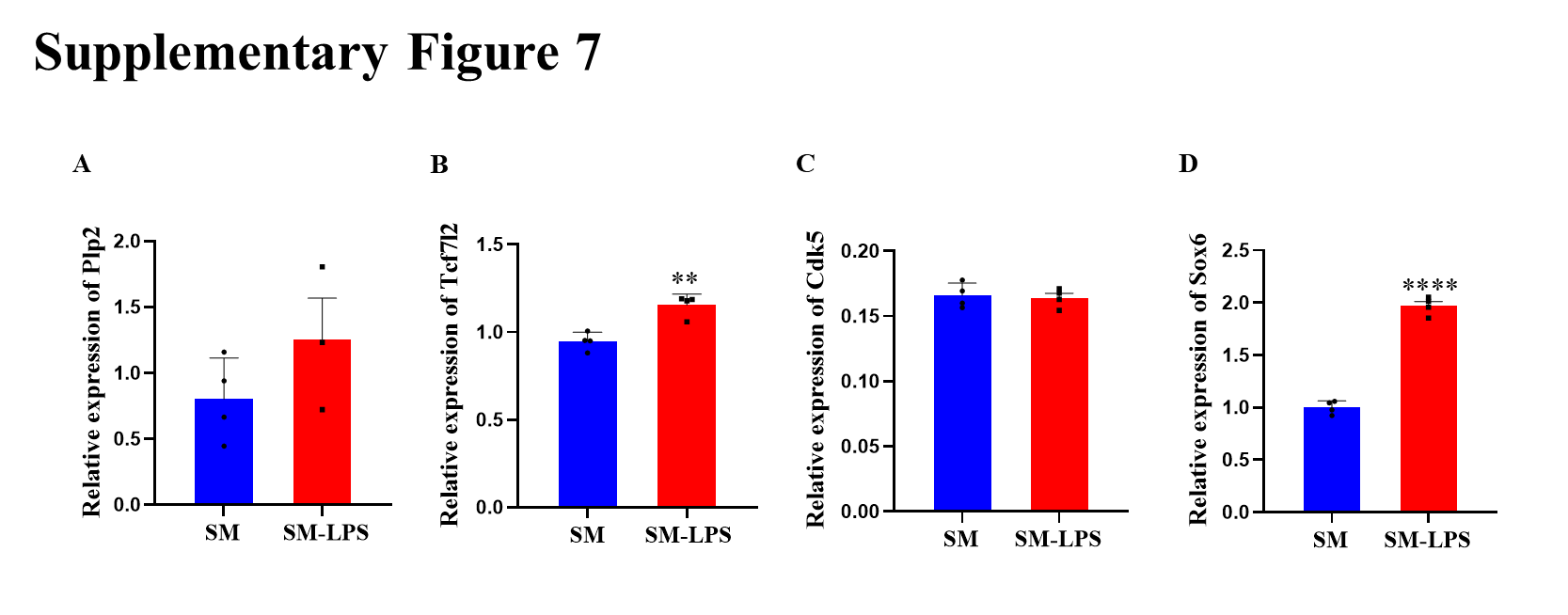
**

**Supplementary Figure 7. Expression of miR-615-5p target genes.**

(A-F) After OPCs were incubated with SM-derived supernatant/SM-LPS-derived supernatant, qRT-PCR was used to detect the other target genes of miR-615-5p, including Plp2, Tcf7l2, Cdk5, Sox6. T-test was used to determine *P* values. ***P* < 0.01, *****P*＜0.0001. One representative of three independent experiments is shown.

**
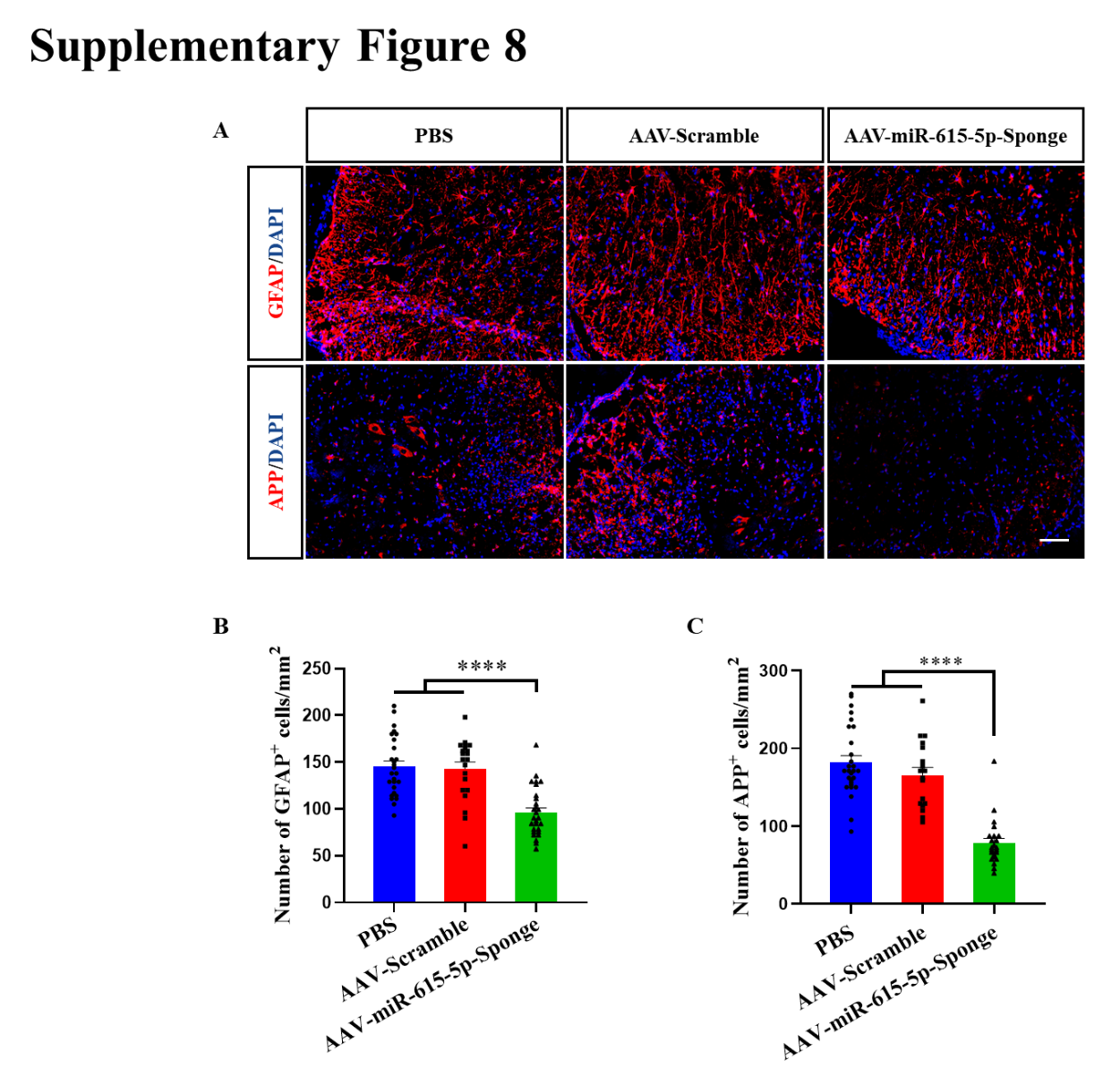
**

**Supplementary Figure 8. Antagonistic miR-615-5p inhibited the expression of GFAP and APP.**

**(A)** Immunofluorescence staining of GFAP and APP in spinal cords of naïve and EAE mice, and **(B, C)** the number of GFAP^+^ cells and APP^+^ cells per field. Scale bar = 100 μm. All data are represented by mean ± SEM (n = 10, each group). One-way ANOVA was used to determine *P* values (B, C). ****p＜0.0001. One representative of three independent experiments is shown.

**Supplementary Table 1** Primers for PCR

| Name | Sequence (5’-3’) |
| --- | --- |
| MYRF 3'-UTR F | ACTGGATCCTGTGTTACACTGGAGCCTGC |
| MYRF 3'-UTR R | CTTGCGGCCGCCCCAACTCACACTGAGGACC |
| Mut MYRF 3'-UTR F | CCTCTTAAAACCTGGCTGCTGGGGCATGAGTCCACGCAGAGT |
| Mut MYRF 3'-UTR R | ACCTCGGCGCCGGGGAGGATGTACGTGATC ATGTCCCGGAGGT |

**Supplementary Table 2** Primers for qRT-PCR

| Name | Sequence (5’-3’) |
| --- | --- |
| miR-615-5p F | GGGGGTCCCCGGTGCTC |
| miR-615-5p R | Reverse Prime from miRcute Plus miRNA qPCR Kit (TianGen) |
| U6 F | CGCTTCGGCAGCACATATAC |
| U6 R | AAAATATGGAACGCTTCACGA |
| MYRF F | GCCAATAAGTCATCGCCAGC |
| MYRF R | GCATCCACCAGGTCCTCTTC |
| MBP F | GCCTGTCCCTCAGCAGATTT |
| MBP R | GTCGTAGGCCCCCTTGAATC |
| Plp2 F | CAGGGACTCCAGTACACCA |
| Plp2 R | GGGATCTGAAGAAGTCAGTCCA |
| Tcf7l2 F | ACAAAGTACCGGTGGTGCAA |
| Tcf7l2 R | AGGCTGACCTTGCTGTGGTA |
| Cdk5 F | AGGGACCTGTTGCAGAACCTA |
| Cdk5 R | ACTGGGGTTCAGAGAGCCTAT |
| Sox6 F | CCCTCTGTCACTGTCACGTT |
| Sox6 R | GCTGTCCCAGTCAGCATCTT |
| GADPH F | CCAATGTGTCCGTCGTGGATCT |
| GADPH R | GTTGAAGTCGCAGGAGACAACC |

**Supplementary Table 3** miR-615-5p target gene network expression table

| Signaling Pathway | Target gene |
| --- | --- |
| Intracellular signal transduction | Src  Smad2  Bcr  Rab40c  Prex1  Deptor  Arhgef12  Asb2  Rasgrp3  Pak6  Syk  Csk  Ksr2  Unc13a  Rgs6  Rps6ka1  Drd2  Nfam1  Myo9b  Gucy2e  Acot11  Cit  Adcy6  Cnih2 |
| MAPK signaling pathway | Nfatc2  Rps6ka1  Rac2  Rasgrp3  Pla2g6  Fgf15  Ppp5c  Mras  Prkacb  Tab1  Map2k2  Pla2g2f  Map3k11  Fgf12  Cacng2  Nr4a1 |
| Neurotrophin signaling pathway | Sort1  Map2k2  Pik3r2  Irs1  Kidins220  Foxo3  Csk  Rps6ka1 |
| Nervous system development | Kat2a  Amigo1  Gfra2  Sema7a  Vegfa  Sema6b  Bex1  Slit3  Mafk  Pppr9b  Mark4  Elavl3  Fev  Insc  Cit  Hmx3  Atcay  Trnp1  Fzd3  Sema4c  Ephb2  Jup  Cplx2 |
| Cell differentiation | Flt3  Cit  Sema4c  Pappa  Vegfa  Sema6b  Insc  Slit3  Ggnbp1  Ptk2b  Tsnax  Sema7a  Zbtb7b  Bex1  Fev  Sort1  Hip1  Ppp1r9b  Csk  Onecut3  Myrf  Elavl3  Hmx3  Bmp3  Amigo1  Cplx2  Ago2  Zbtb7a  Agrn  Abhd5  Hand2 |
| Multicellular organismal development | Nrarp  Foxc2  Meis2  Tbx2  Agrn  Fzd8  Snai1  Mbnl3  Sort1  Bex1  Bmp3  Celsr2  Wnt2  Hmx3  Slit3  Ppp1r9b  Dact2  Tbx5  Amigo1  Zbtb7b  Sema7a  Ggnbp1  Ephb2  Sema6b  Insc  Ago2  Aida  Sema4c  Trnp1  Casz1  Chrd  Vdr  Wnt9b  Fzd3  Elavl3  Cit  Spry4  Unc5a  Spred3  Tsnax  Wnt2b  Vegfa  Zbtb7a  Fev |
